# Conformational perturbation, allosteric modulation of cellular signaling pathways, and disease in P23H rhodopsin

**DOI:** 10.1038/s41598-020-59583-2

**Published:** 2020-02-14

**Authors:** Kristina N. Woods, Jürgen Pfeffer

**Affiliations:** 1 Lehrstuhl für BioMolekulare Optik, Ludwig-Maximilians-Universität, 80538 München, Germany; 2 Technical University of Munich, Bavarian School of Public Policy, 80333 München, Germany

**Keywords:** Biophysics, Computational biology and bioinformatics

## Abstract

In this investigation we use THz spectroscopy and MD simulation to study the functional dynamics and conformational stability of P23H rhodopsin. The P23H mutation of rod opsin is the most common cause of human binding autosomal dominant retinitis pigmentosa (ADRP), but the precise mechanism by which this mutation leads to photoreceptor cell degeneration has not yet been elucidated. Our measurements confirm conformational instability in the global modes of the receptor and an active-state that uncouples the torsional dynamics of the retinal with protein functional modes, indicating inefficient signaling in P23H and a drastically altered mechanism of activation when contrasted with the wild-type receptor. Further, our MD simulations indicate that P23H rhodopsin is not functional as a monomer but rather, due to the instability of the mutant receptor, preferentially adopts a specific homodimerization motif. The preferred homodimer configuration induces structural changes in the receptor tertiary structure that reduces the affinity of the receptor for the retinal and significantly modifies the interactions of the Meta-II signaling state. We conjecture that the formation of the specific dimerization motif of P23H rhodopsin represents a cellular-wide signaling perturbation that is directly tied with the mechanism of P23H disease pathogenesis. Our results also support a direct role for rhodopsin P23H dimerization in photoreceptor rod death.

## Introduction

Retinitis pigmentosa (RP) is a progressive retinal degenerative disease caused by a heterogeneous genetic defects that affect more than a million people worldwide. The disease is characterized by initial night blindness and then a progressive loss of peripheral vision due to rod photoreceptor cell death^1^.

More than 100 rhodopsin mutations are associated with RP and approximately 20–40% of RP cases are autosomal dominant RP (ADRP)^2^. P23H *RHO* is the first and most frequently reported mutation in ADRP cases and for this reason has been extensively studied in cellular and animal models to evaluate its pathobiology. Two classes of opsin mutations have been designated based on studies in tissue culture cells^3^. Class 1 opsin mutants are similar to wild-type (WT) rhodopsin in expression levels, fold correctly, and form a functional photoreceptor. Class 2 opsin mutants on the other hand exhibit low expression levels, possess a decreased ability to regenerate the retinal, and have inefficient transport to the plasma membrane. The P23H mutation in rhodopsin is an example of a class 2 mutation.

There is a strong consensus that the P23H RHO mutation is linked with ADRP^4^. Although, the specific role of the P23H mutation itself in the disease pathology is unclear. One early hypothesis suggested that P23H-induced unfolded protein response (UPR) is the major determinant in triggering photoreceptor death^4^. Early studies reported that P23H misfolded protein is retained in the ER and not transported to the cell membrane but rather degraded by the ubiquitin-proteosome system^1^. Co-expression of P23H and WT opsin was found to result in enhanced proteasome mediated degradation of both mutant and WT opsin, implying that co-aggregation prevented rod outer segment (ROS) formation. The dominant negative effect on ROS formation was interpreted as the underlying cause for RP progression^5^. The results from more recent investigations have begun to revise this interpretation of RP. Newer investigations have established that P23H rhodopsin can escape the ER quality control mechanisms and reach the ROS^6,7^. The escaped P23H forms aggregates that create abnormal internal membrane structures that are destructive to WT receptors. Therefore, in this revised interpretation, it is the toxicity to WT receptors and the gain of function attributes of P23H in the ROS that lead to photoreceptor cell degeneration.

Irrespective of the specific mechanism underlying the progression of RP, it has become increasingly more apparent that the conformational plasticity^4,8^ of P23H is a major contributing factor to the underlying causation of disease. For this reason, in this investigation, we use both THz spectroscopy and MD simulation to characterize the molecular-level functional dynamics and conformational heterogeneity of P23H rhodopsin. Our aim is to gain greater insight into the connection that links the inherent propensity of P23H to adopt a more conformationally flexible structure and the consequent shift in protein interaction dynamics that leads to the disease state.

## Results

### Experimental detection of the conformational stability of P23H rhodopsin

#### Experimental detection of the global motions in dark-state P23H rhodopsin

We first examine the global fluctuations of P23H rhodopsin with THz spectroscopy. The global fluctuations, which reside in the <100 cm^−1^ region of the infrared spectrum, describe the intrinsic dynamics of the receptor^9^. These globally, correlated fluctuations detected reflect the sampling of the ensemble of conformations that comprise the free energy landscape of all possible receptor conformations. Therefore, they provide direct information about the sampling of conformational substates in the mutant receptor. ADRP has been classified as a conformational disease^10^. Hence, a more detailed understanding about the underlying conformational ensemble dynamics of P23H rhodopsin may provide unique insight into the nature and progression of the disease.

**Figure 1:**
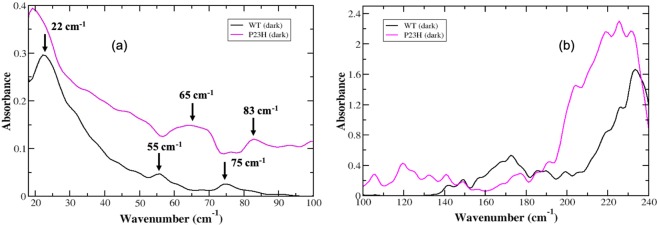
Experimental THz spectrum of the dark-state of WT (black, solid line) and P23H rhodopsin (purple, solid line) in **(a)** the 20–100 cm^−1^ spectral region and in **(b)** the 100–240 cm^−1^ spectral region.

An inspection of the resolved low frequency peaks of P23H rhodopsin in Fig. 1a reveal that modes in the >50 cm^−1^ are noticeably different when contrasted with the wild-type (WT) receptor. Specifically, there is a shift of the prominent peak at 55 cm^−1^ in WT rhodopsin to a blue-shifted, broad peak in P23H rhodopsin centered at 65 cm^−1^. We also observe a 10 cm^−1^ blue-shift of the 75 cm^−1^ peak in WT rhodopsin to 83 cm^−1^ in P23H.

In our previous work on rhodopsin^11^, we determined that the peak at 55 cm^−1^ is associated with a retinal (polyene chain) torsional fluctuation that is coupled with a protein, global backbone torsion. The peak at 75 cm^−1^ is attributed to a retinal torsional oscillation that is coupled with collective out-of-plane protein side-chain fluctuations^11^. Hence, the blue-shift of the two P23H dark-state modes could be attributed to two distinctive mechanisms (*i*) the mutation in the EC region of receptor results in an increase in the strength of the H-bonding and other weak associations between the receptor and retinal in ligand-binding pocket or (*ii*) amino acid residue interactions (protein intramolecular interactions) within the P23H retinal binding pocket are enhanced in response to a weakening of interactions between the retinal and the protein. The latter is in line with previous investigations on P23H rhodopsin expressed in the presence of 9-*cis* retinal^12^. This earlier work demonstrated that P23H rhodopsin bound with 9-*cis* retinal is thermally unstable and is also more rapidly bleached with hydroxylamine in the dark-state when compared with WT receptor. We also observe a small but significant difference in the lowest frequency mode when contrasting the dynamics of the receptors in Fig. 1a. The prominent band at 22 cm^−1^ in WT rhodopsin is slightly red-shifted to 19 cm^−1^ in P23H. This peak is ascribed to the global oscillation of receptor side-chains^13^ and has implications about the stability of the receptor as a whole. The slight red-shift of this lowest frequency band in P23H rhodopsin suggests that the receptor is altogether conformationally more flexible. This is further supported by the overall higher intensity of the spectrum of global modes in P23H. The increased absorption in the ≤ 100 cm^−1^ experimental spectrum indicates that P23H has greater conformational plasticity when compared with WT rhodopsin.

#### Experimental detection of the long-range correlated fluctuations in P23H rhodopsin

Experimentally, we detect more localized segmental motions in the receptor (i.e. receptor dynamics comprising a subset of the molecule rather than the entire receptor) in the 100–250 cm^−1^ spectral region. Motions detected in this higher frequency region are sensitive to local relaxations that reflect specific intramolecular and intermolecular induced correlated fluctuations. In the >100 cm^−1^ spectral region of P23H in Fig. 1b we observe noticeable differences in these motions when compared with the WT receptor. Particularly, it becomes more apparent from the higher frequency spectrum that the P23H receptor is relatively (thermally) unstable. The numerous additional peaks in P23H between 100 cm^−1^–135 cm^−1^ in Fig. 1b point to *uncorrelated* fluctuations taking place within the 3-D receptor structure. There is also a clear decrease in the absorption intensity of the P23H modes in the 160–180 cm^−1^ spectral region. The 160–180 cm^−1^ spectral region is related to solvent shell dynamics and solvent-protein coupled motions^14^. The reduced intensity of the P23H absorption spectrum in this region indicates that that there is a decrease in the solvent and solvent-coupled interhelical H-bonding network in P23H. This breakdown of the extensive H-bonding network in the receptor would probably lead to two things (*i*) loss of structural stability in the receptor and/or (*ii*) misfolding due to interhelical contacts that would form to take the place of the absent solvent-mediated H-bonding network of interactions. The >180 cm^−1^ of the spectral region is dominated by a protein-coupled – retinal torsional mode involving the C_11_ = C_12_ torsion angle of the retinal^15^. The protein-retinal torsional mode is centered at 230 cm^−1^ in the WT rhodopsin experimental spectrum. In Fig. 1b, we find that this mode in the P23H spectrum is both red-shifted and broadened suggesting that the retinal has weaker interactions with the protein residues in the ligand-binding pocket.

#### Experimentally detected global motions in Meta-II-P23H

**Figure 2:**
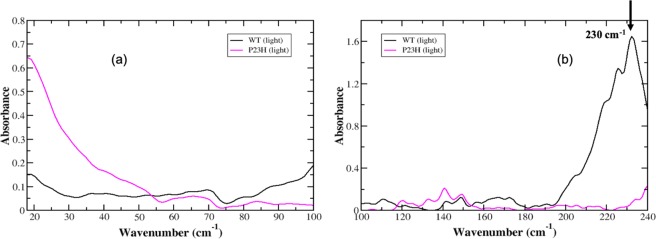
Experimental THz spectrum of the dark-state of WT (black, solid line) and P23H rhodopsin 776 (purple, solid line) in **(a)** the 20–100 cm^−1^ spectral region and in **(b)** the 100–240 cm^−1^ spectral region.

As stated previously, the dark-state global mode spectrum of P23H rhodopsin features three major detectable peaks in the 20–100 cm^−1^ spectral region. There is a peak at 19 cm^−1^, a broad band centered close to 65 cm^−1^ and a higher frequency peak at approximately 85 cm^−1^. Upon light activation both of the higher frequency modes decrease slightly in intensity and there are no other prominent modes that emerge after isomerization has taken place (Fig. 2a). Additionally, the dark-state P23H mode centered at 19 cm^−1^ in Fig. 1a is also slightly red-shifted in the Meta-II-P23H spectrum in Fig. 2a. The lack of changes in the P23H global mode spectrum following isomerization is both surprising and curious. In general, one would expect noticeable changes in the global modes of the receptor that reflect changes in the retinal environment that accompany isomerization – such as those observed in the WT spectrum. The coupling of retinal changes to receptor global structural transitions is important for stabilizing the active-state receptor conformation^16–18^. In our previous experimental investigation on WT rhodopsin we detected distinct changes in the global motions of the receptor that reflected the adaptation of receptor dynamics to localized changes in the ligand-binding pocket that were ultimately transmitted to the rest of the receptor during activation.

#### Experimentally detected correlated structural fluctuations in Meta-II-P23H

In the light-state of P23H rhodopsin (Meta-II) the more localized spectrum of correlated motions more closely resembles that of the WT spectrum in Fig. 2b, particularly in the 100–150 cm^−1^ spectral region. In the WT Meta-II receptor, we have previously^11^ identified functionally relevant peaks at 110 cm^−1^, 120 cm^−1^ and 130 cm^−1^. These peaks are associated with anharmonic, solvent-mediated fluctuations that couple to protein main-chain and backbone atoms, respectively and are presumed to be essential in the reaction coordinate of the isomerization process. Peaks in the WT spectrum at approximately 150 cm^−1^ and 140 cm^−1^ (Fig. 2b) are associated with interhelical and solvent-induced H-bonding interactions that mediate structural stability in the receptor core. In the Meta-II-P23H spectrum in Fig. 2b, we detect an overall decrease in mode intensity in the 100–135 cm^−1^ spectral region when compared with the dark-state P23H spectrum. We also observe a reduction in the number of additional (dissimilar) modes in this region when contrasted with the WT Meta-II spectrum. The decrease in mode intensity could denote a more stable tertiary structure in the light-state receptor when compared with the dark-state. Although, the decrease in the number of peaks could also be an indication that there are structural aberrations due to weak or unstable intra-protein interactions that destabilize the dynamics of the entire receptor – and hence, reduce the number of overall modes. The latter hypothesis seems to be a more reasonable interpretation of the P23H experimental spectrum. For example we note an almost complete absence of spectral intensity in the 160–180 cm^−1^ region in the P23H spectrum when contrasted with the WT receptor in the same region. As mentioned previously, this spectral region is associated with the solvation shell network of H-bonding interactions. The lack of intensity in the P23H spectrum in this region suggests that the receptor solvation shell is significantly disrupted during/after the isomerization process. The displacement of water molecules in the protein solvation shell would likely promote misfolding in the receptor structure by increasing the number of incorrect protein intra/interhelical H-bonds to compensate for the lost network of solvent interactions. Interestingly, in the >200 cm^−1^ spectral region, we observe the loss of the 230 cm^−1^ retinal C_11_ = C_12_ torsional mode in the P23H spectrum. Recent femtosecond time-scale experiments on rhodopsin^19^ have indicated that this particular retinal torsional oscillation is central in the process involved with transferring energy from the retinal to the protein during isomerization. The observation that this peak is entirely missing from the P23H spectrum indicates that the activation mechanism is altered when compared with the WT receptor.

### MD simulation of P23H rhodopsin

#### Mutation and the effects on long-range communication pathways in P23H rhodopsin

**Figure 3:**
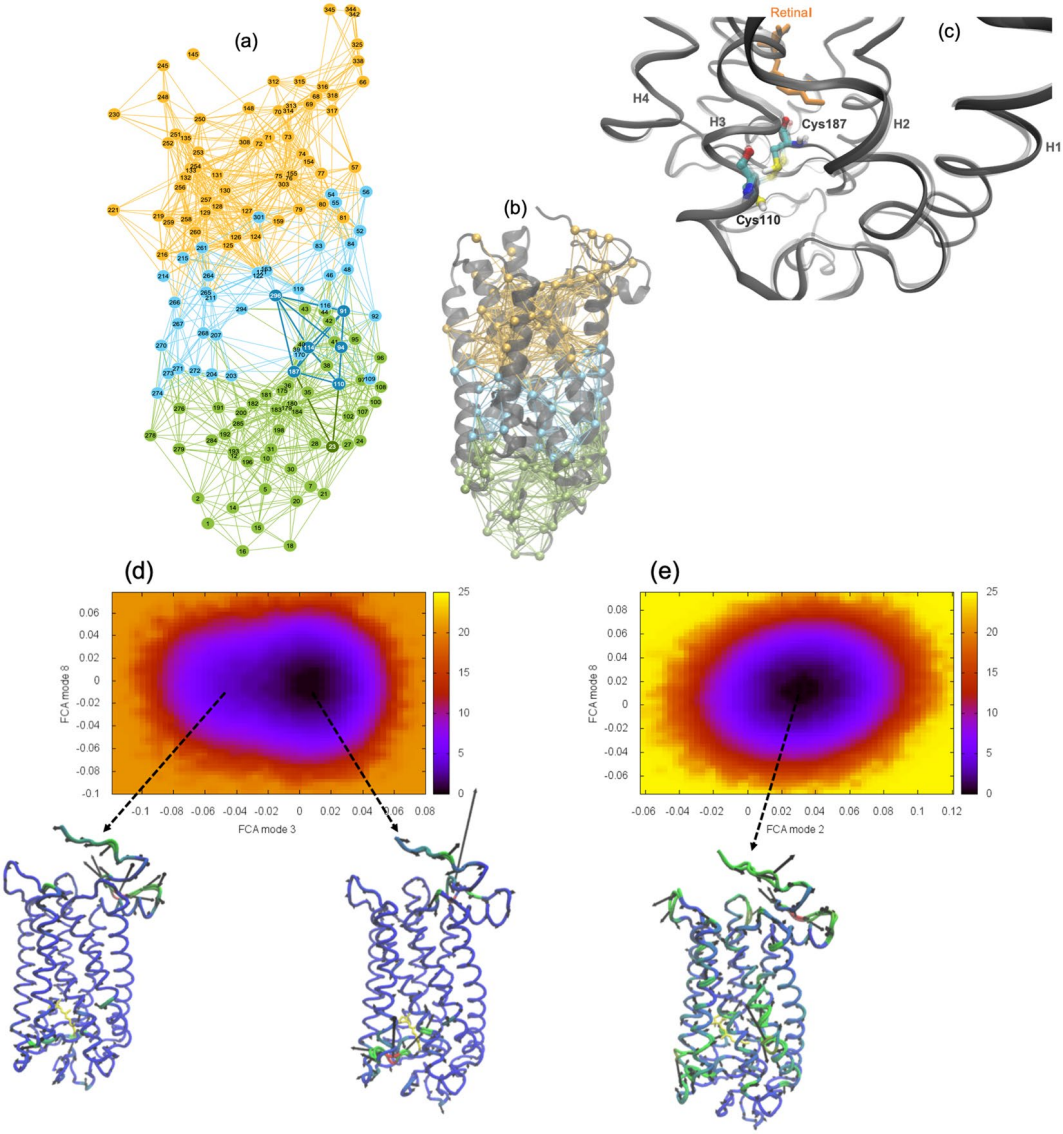
(**a**) Localized structural fluctuation (LSF) network of interactions from a MD simulation of P23H rhodopsin in the dark, inactive-state. In the network representation of the LSFs the nodes represent C-α amino acid residues and the links between the nodes represent localized interactions. In the network representation of the LSF, we find that the connections in the inactive receptor are grouped into three separate communities. (**b**) The corresponding LSF is mapped onto a 3-D cartoon representation of P23H rhodopsin. (**c**) Overlay ribbon diagram representation of the extracellular (EC) backbone of dark-state WT rhodopsin (gray, transparent) and P23H rhodopsin (gray). A ball-and-stick representation of WT (transparent) and P23H (CPK coloring) residues Cys110 and Cys187 are also displayed showing the ruptured bond in P23H when compared with the WT receptor. Free energy surfaces derived from the full correlation analyses (FCA) of the MD trajectories of (**d**) WT and (**e**) P23H rhodopsin. The C-α representation of the rhodopsin molecules illustrate the dominant motion within the minimum of the energy surfaces where regions colored in red show greater mobility and regions in blue have less mobility.

We have also carried out MD simulations on WT and P23H rhodopsin. One of the advantages of computational simulation is that it allows us to directly analyze the intramolecular and intermolecular interactions that are altered with mutation. And this in turn provides us with deeper insight into the underlying mechanisms that may alter the conformational ensemble dynamics and receptor interaction networks that we directly probe with experiment. An analysis of the MD simulation induced localized structural fluctuations (LSFs) in P23H dark-state rhodopsin reveals that mutation disrupts the long-range correlations in the receptor (Fig. 3a,b). A mapping of the LSFs of P23H in Fig. 3 illustrate that the receptor dynamics is divided into two distinct domains. And this loss of long-range interactions ultimately translates as large-scale, uncorrelated motion in various parts of the receptor (Supplementary Figure S1). The residues at the interface of the domains are those primarily associated with the initiation and propagation of signal transduction in the activation process of the receptor. For instance, in Fig. 3a it becomes more apparent from examining the network of interactions in P23H rhodopsin that the mutation at residue 23 (His23) is directly connected with localized fluctuations that disrupt the extended H-bonding network environment involving Glu181, Ser186, and Tyr268 that stabilize the retinal protonated Schiff base (PSB). This disruption of H-bonding interactions directly results in a physical deformation of the receptor ligand binding site (Supplementary information, Section 1 and Supplementary Figure S2) that leads to the rupture of the Cys110–Cys187 disulfide bond in the extracellular, intradiscal domain (Fig. 3c). Consequently, the motion of the retinal and the residues surrounding the retinal pocket become thermally unstable. In effect, the (localized) fluctuations of the residues in the ligand-binding region become uncorrelated with the overall global dynamics of the receptor. Accordingly, signal transmission from the retinal to the rest of the receptor is also hindered. The P23H mutation, although confined in the N-terminus of the receptor, has long-range effects in both the global structure and overall dynamics of the receptor. The mutation predominately disrupts the extended network of H-bonds in the ligand-binding region that are known to regulate the activity of the retinal, but these altered (localized) structural fluctuations also appear to have a causal effect on the global motions of the receptor as a whole.

#### Conformational landscape and P23H rhodopsin potential for activation

We have used MD simulation to analyze the internal protein motions of dark-state WT and P23H rhodopsin. There are a growing list of both computational and experimental studies that have demonstrated that protein landscapes are comprised of conformational (sub)states^20–24^ with dynamical and structural characteristics that describe the distinctive function of the protein. In the native state, a protein exists as an ensemble of interconverting conformations driven by thermal fluctuations. Internal protein motions correspond to the interconversion of protein conformations as they move within a conformational (sub)state or as they move from one conformational state to another. The possible (conformational) transitions within this ensemble of conformations describes the protein conformational landscape. And the sampling of conformational populations within this pre-existing ensemble provides insight into the mechanism of protein function. In this study, we have used full correlation analysis (FCA) of protein dynamics from MD simulations to map out the conformational landscape of P23H and WT rhodopsin. Our aim is to form a deeper understanding about the role of conformational sampling in P23H rhodopsin and the manner in which this may influence receptor function.

In Fig. 3d, we find that the FCA of correlated motions in WT rhodopsin reveals an energy landscape consisting of two major conformational states. The conformational states are identified as minima on the plotted free energy surface. The two minima of unequal energy (a deep basin and a shallow basin) are connected by a low energy barrier. Analysis of the dynamics of the rhodopsin structure within the deeper energy basin uncovers a collective torsional oscillation that involves backbone motion of all the TM helices. The collective backbone motion connects the EC side of rhodopsin with the G-protein coupled region and has the largest amplitude dynamics near the retinal pocket. We identify this collective torsion with the principal motion of the dark-state receptor from MD simulations of WT rhodopsin. The dynamics of rhodopsin in the shallower well closely resembles the primary motion of the WT active-state (Meta-II) receptor from MD simulation. The active-state-like dynamics can be described as an elongation torsion that compresses inner core residues on helix H3 (residues Leu119–Glu122) and creates a correlated set of structural fluctuations that couple regions of the N-terminus, the (intracellular loop 2) IL2 loop between helices H3 and H4, and the C-terminus. Hence, the dark-state WT receptor reveals a conformational landscape that samples from both inactive- and active-state type dynamics. Subsequent examination of the transition pathway (Supplementary information, Section 2A and Supplementary Figure S3) between the two conformational states reveals that the WT receptor occupies the deeper energy basin for the majority of simulation time but makes frequent, transient transitions to the shallower basin by using a correlated stretching motion of the N- and C-terminal regions to facilitate the crossing over the low energy barrier.

The WT rhodopsin correlation analysis is in line with our previous work on rhodopsin^11^. Using an entirely different approach that focused on probing the intra-protein structural fluctuations within rhodopsin, we deduced that there is an equilibrium of both inactive and active-state protein conformational fluctuations in the WT dark-state protein. We construed that conformational heterogeneity in the inactive receptor indicates that rhodopsin samples a diverse set of functional structures even before any activation event has taken place.

The same correlation analysis conducted on P23H rhodopsin in Fig. 3e exposes a drastically different conformational landscape. The P23H inactive receptor has only a single energy well. An exploration of the receptor dynamics within the energy well reveals fragmented, uncorrelated fluctuations throughout the entire receptor (Fig. 3e). Using the WT receptor for comparison, the analysis of correlated motion in the P23H receptor indicates that the mutation somehow obstructs the activation process in rhodopsin.

### MD simulation of meta-II-P23H rhodopsin

#### Meta-II-P23H, misfolding, and the increased propensity for forming specific dimerization motifs

In the previous section, our analysis of P23H correlated motions from MD simulation indicated that the receptor would likely not be able to transition to the activated Meta-II state. Despite our findings (including Supplementary information, Section 4 and Supplementary Figures S16,S17), there are multiple experimental accounts that report (that although altered from the WT receptor) that P23H rhodopsin does transition to a functioning Meta-II state^4,25^. Computationally, we are unable to actively map (in real time) the transition from inactive- to active-state rhodopsin. Therefore, we have elected to introduce the P23H mutation to the crystal structure of WT Meta-II rhodopsin and document the changes in structure and dynamics that occur with mutation as a function of MD simulation time.

**Figure 4:**
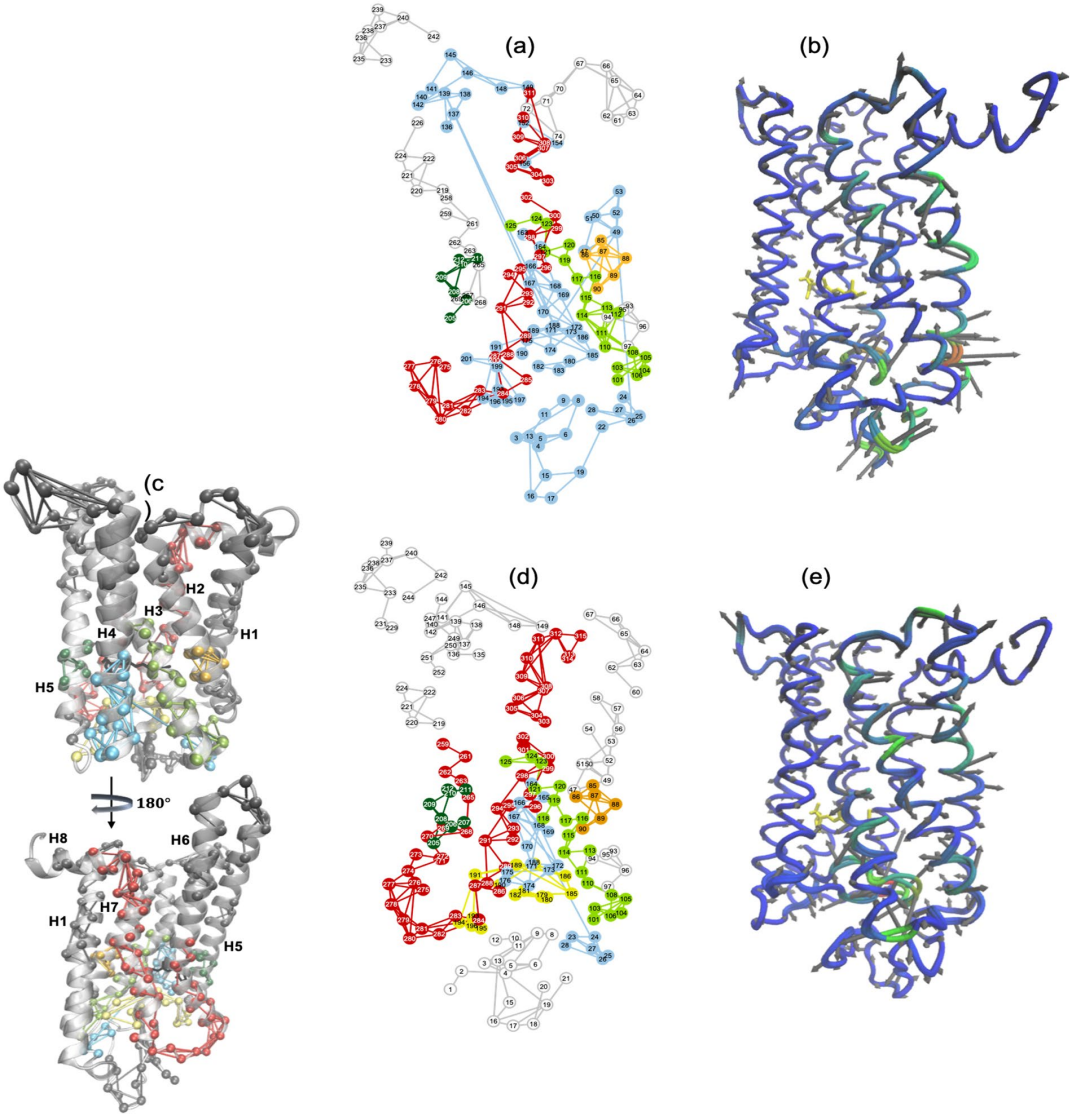
(**a**) A 2-D mapping of the Meta-II-P23H LSF from MD simulation. The different colors in the network mapping of the LSFs represent regions of correlated fluctuations. (**b**) A C-α representation of the dominant PCA mode (PCA1) of Meta -II-P23H from MD simulation. Regions colored in blue represent areas of less mobility and regions in red illustrate regions with more mobility. (**c**) Cartoon representations of Meta-II rhodopsin showing the mapping of the Meta-II LSF from the MD simulation onto the rhodopsin 3-D structure. (**d**) A 2-D mapping of the Meta-II LSF and (**e**) the C-α representation of the PCA1 of Meta-II from MD simulation.

Consistent with the dark-state receptor, our LSF analysis of Meta-II-P23H in Fig. 4a reveals that the ruptured Cys110–Cys187 disulfide bond in the extracellular loop 2 (EL2) region is the source of extreme misfolding throughout the entire receptor. Predominantly, strong correlations between residues in the EC regions of helix H4 (Ala164–Pro170), helix H1 (Leu47–Pro53) and EL2 lead to severe compression of the retinal-binding site. As a result, the extracellular (EC) and N-terminus region of the P23H active-state receptor become structurally fluid/unfolded (Fig. 4b). Further, we note that the exodus of many of the conserved water molecules in the receptor core disrupt the stability of many of the structural motifs that are important for WT receptor activation (Fig. 4c,e). For example, close interaction between Met207 and the β-ionone ring on one side of the retinal and irregular interactions between Gly89–Gly90 (TM2) and Glu114 (TM3) adjacent to the polyene chain interfere with the concerted “switching” of functional microdomains that link volume/shape changes in the active-state ligand-binding site with rearrangements in the packing density of transmembrane helices (TM2, TM3, TM6, and TM7). These particular core packing density readjustments in Meta-II form a signaling channel from the ligand-binding site to the cytoplasmic side of the receptor (Fig. 4c,d). Disruptions in membrane packing also impede H-bonding interactions that form structural constraints that stabilize the intracellular (IC) side of the active-state receptor. We find that in Meta-II-P23H, disruption in solvent-mediated protein interactions hamper the formation of the network of H-bonds that shape the conserved NPxxY microdomain located at the cytosolic side of TM7. The conserved Tyr223 (TM5) interaction with Leu132 (TM3) is also weakened by hydrophobic packing abnormalities that form as a result of the altered EL2 conformation in Meta-II-P23H. The TM3–TM5 interfacial association, coupled with structural changes in EL2, stabilize helix H5 in an active orientation in WT Meta-II^26^.

We have also identified that the distortion of the transmembrane core in Meta-II-P23H may also underlie the receptor potential for *specific* dimerization. For example, the deformation of the retinal-binding region exposes a stable interface for the formation of the well-known H1/H8–H1/H8 rhodopsin dimer^27,28^. Explicitly, aromatic residues on helix H1 such as Phe45, Leu49, and Phe52 along with EL1 residues like Tyr96 and His100 are made more accessible with the receptor distortion (Fig. 4a). The exposed residues create a well-defined network of interactions such that the potential to create a stable receptor-receptor interface is likely increased in the mutant receptor. Structurally, we also note an increase in the β-sheet content, particularly in EL1 and EL3 and a reduction in the α-helical content of the receptor predominately at the EC ends of helices H5 and H7. This supports earlier experimental FTIR spectromicroscopy investigations^29^ obtained from P23H in cells. From the FTIR spectromicroscopy studies, it was concluded that the increase in β-sheet structure and decrease in α-helical structure observed in P23H may be a general feature in proteins with destabilizing mutations that are prone to misfolding and *specific* aggregation. We have also noted other mutation-induced structural and dynamical alterations in Meta-II-P23H that suggest that other types of dimerization structures may also be made more accessible in the mutant receptor but this topic will be addressed in greater detail in a future section.

#### Receptor-receptor self-association in WT and P23H rhodopsin

There is growing evidence that GPCR homodimerization, heterodimerization and general oligomeric assembly may have important functional roles^30–32^. In fact, protein self-association has been hypothesized to be central in P23H rhodopsin disease pathogenesis^6,33,34^. In our own analyses of the internal dynamics of Meta-II-P23H we observed that distinct alterations in P23H dynamics may also be linked with an increased potential for *specific* receptor-receptor contacts. For these reasons we have conducted multiple self-assembly coarse-grained (CG) MD simulations of model membranes containing 16 rhodopsin molecules. Our aim is to gain a better understanding of how modified structural and inter-residue dynamics in P23H rhodopsin may promote distinct types of receptor interfaces that facilitate oligomerization.

**Figure 5:**
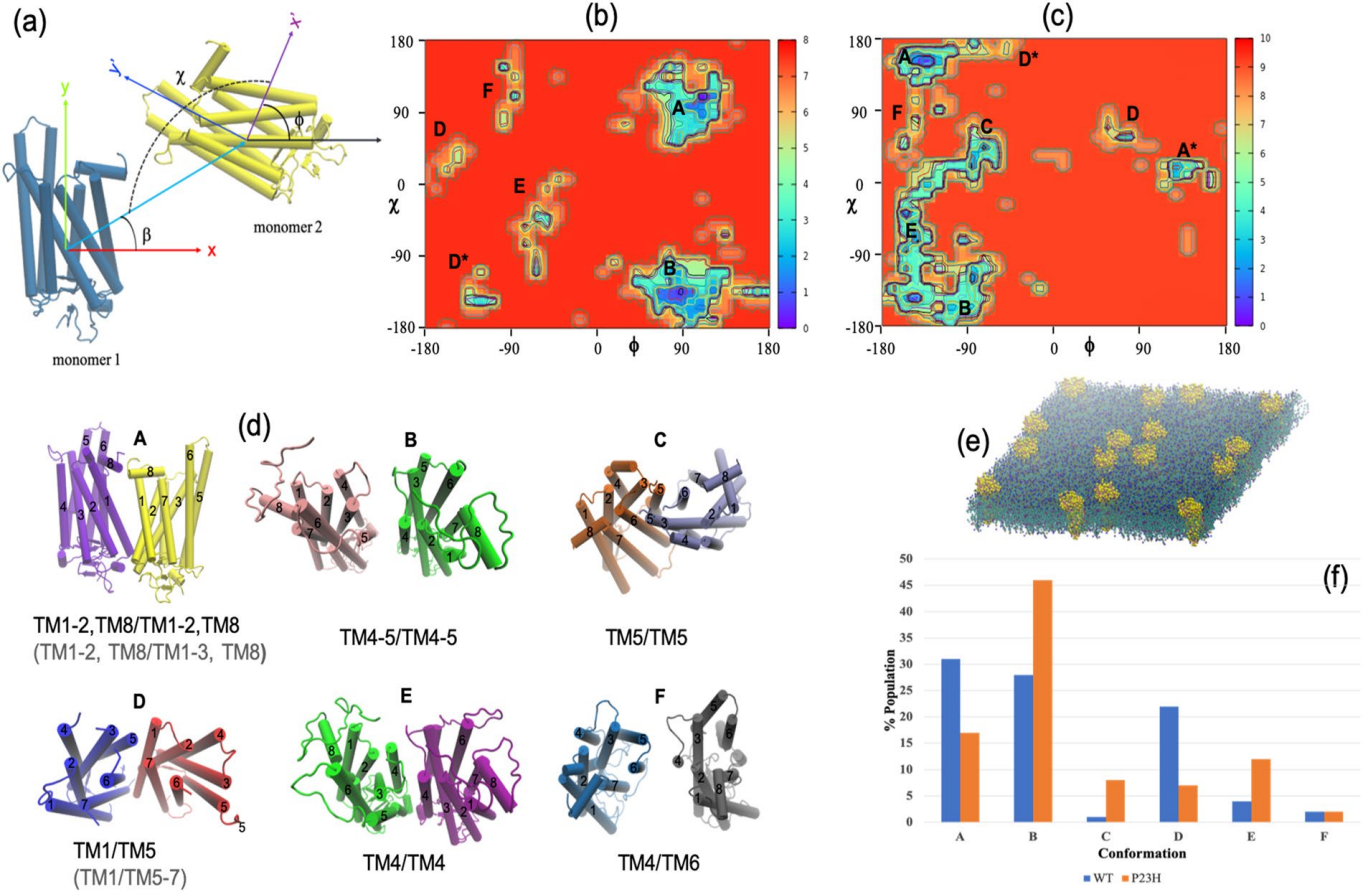
(**a**) Angles used to describe the orientation of the monomers within the receptor dimers. Explicitly, β describes the position of monomer 2 with respect to monomer 1, χ is the angle that designates the contact orientation between monomers 1 and 2, and ϕ is the rotation of monomer 2 about its z-axis. The sampled (**b**) WT and (**c**) P23H homodimer configurations from the CG simulations. (**d**) A cartoon representation of the dimer configurations as labeled in (**b**) and **(c**). The labeling in gray refers to the slightly different dimer structures in the P23H self-assembly homodimer system when compared with the WT rhodopsin self-assembly system. (**e**) A typical early timescale arrangement of the self-assembly simulations comprising 16 receptors. (**f**) Relative populations of the dimer configurations found in the respective CGMD self-assembly simulations.

Conformational analysis of the arrangement of rhodopsin dimers formed in the self-assembly simulations of separate WT and P23H rhodopsin molecules have uncovered unexpected details about the role that mutation may play in rhodopsin self-association (Fig. 5a). The WT dimer configurations were similar to those uncovered in previous work^35^ on CG MD simulations of rhodopsin dimerization. Briefly, we found six prominent dimer types that form in the self-assembly simulations as illustrated in Fig. 5b. The preferred and most stable WT rhodopsin dimer exists in a configuration where residues on transmembrane helices H1, H2 interact on the extracellular side and residues on (the amphipathic) helix H8 interact at the cytoplasmic surfaces. This has been referred to as the H1/H2/H8–H1/H2/H8 dimer. The second most prominent WT dimer is the H4/H5–H4/H5 dimer. The H4/H5–H4/H5 dimer interface is supported by direct interactions between residues on EL2 and IL3 and (indirect) lipid-mediated interactions between residues on helices H4 and H5. The H4/H5–H4/H5 arrangement has weaker inter-receptor residue connections when compared with the H1/H2/H8–H1/H2/H8 dimer. Other prominent dimer types that form in the WT self-assembly system include a dimer that connects residues on TM1 on one receptor with residues on TM5 on the other receptor (H1/H5) as well as the somewhat less encountered dimers H4/H4 and H4/H6.

Intriguingly, we only identify two *prominen*t dimer types in the P23H self-assembly simulations (Fig. 5c,f). As with the WT, the H1/H2/H8–H1/H2/H8 dimer represents the lowest energy dimer formed between interacting receptors but it does not represent the most prominent dimer. Rather, an average over the group of simulations indicates that the preferred dimer type amongst the P23H receptors is the H4/H5–H4/H5 dimer. The H4/H5–H4/H5 dimer arrangement is adopted almost 50% of the time (Fig. 5f). It is also worth noting that the H4/H5–H4/H5 dimer has lower energy than the corresponding dimer type in the WT system and also a significantly broadened conformational spread (Fig. 5c). This is likely due to the more flexible ECD in P23H that accordingly permits stronger residue interactions at the EC dimer interface but more flexibility in the transmembrane and cytoplasmic regions. For instance, we detected that during the course of the P23H self-assembly simulations that an established H4/H5–H4/H5 dimer would often intermittently adopt a H4/H4 or H5/H5 dimer conformation for short periods of time (a few to tens of nanoseconds) before returning back to the original H4/H5–H4/H5 dimer conformation. Given these results, it is interesting to consider if the dominance of a particular dimer type in P23H rhodopsin has any particular functional relevance?

### MD simulation of rhodopsin dimers and oligomers

#### Receptor dimerization and an altered conformational landscape in rhodopsin: the H1/H2/H8–H1/H2/H8 dimer

Since the emphasis of this investigation is on P23H rhodopsin, we will limit our discussion to the principal dimer configurations uncovered in the P23H rhodopsin self-assembly simulations. Our aim is to determine if the top dimerization motifs discovered have a functional role in P23H and/or WT rhodopsin. Towards this end, we have converted the CG systems back to an all-atom representation. And have subsequently conducted atomic-level MD simulations on the individual dimers.

**Figure 6:**
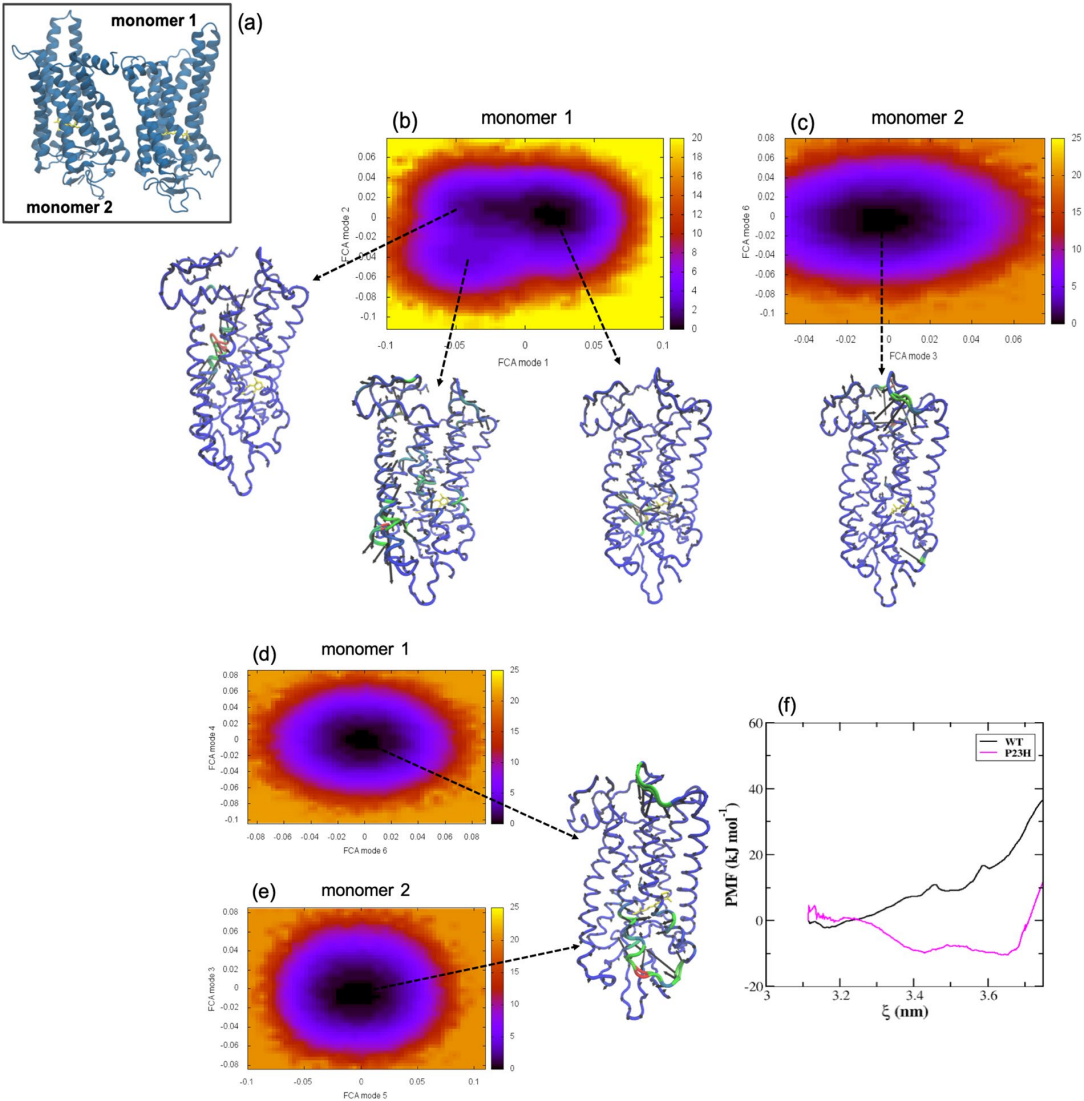
(**a**) Carton representation of rhodopsin molecules illustrating the arrangement of the receptors in the H1/H2/H8–H1/H2/H8 dimer. Free energy surfaces derived from the full correlation analyses (FCA) of the MD trajectories of (**b**) rhodopsin M1 and (**c**) rhodopsin M2 forming the WT H1/H2/H8–H1/H2/H8 dimer. And the FCA of P23H rhodopsin (**d**) M1 and (**e**) M2 from the MD simulations of the P23H H1/H2/H8–H1/H2/H8 dimer. The C−α representations of rhodopsin shows the principal motion within the minimum of the free energy surfaces. (**f**) The potential of mean force (PMF) in the inter-receptor binding region of the WT H1/H2/H8–H1/H2/H8 WT dimer (black, solid line) and the P23H H1/H2/H8–H1/H2/H8 dimer (magenta, solid line).

In the WT receptor, we find that the H1/H2/H8–H1/H2/H8 dimer (Fig. 6) affects the conformational landscape and therefore, possibly receptor function. The analysis of correlated motions in one of the monomers of the WT dimer (monomer 1 or M1) reveals two conformational states of unequal energy (Fig. 6b) – but there is also an additional, new state in the conformational landscape that was not found in the WT monomer (Fig. 3d). The deepest energy basin of M1 is associated with the receptor “dark-state-like” dynamics that is analogous to what was observed in inactive (WT monomer) rhodopsin. Likewise, the second major energy well is associated with more “active-state-like” fluctuations. The new energy state that appears in M1 reflects new dynamics associated with the coupling of the two receptors and is mainly localized to the EC regions of helices H2 and H3 near the retinal Schiff base. Particularly, the dynamics involves Gly89, Gly90, and Gly114 which are the residues in helices H2 and H3 that are brought close to the Schiff base counterion during activation. As with the WT monomer, the receptor spends most of the simulation time in the dark-like-state substate and transiently samples the more active-like protein conformation (Supplementary information, Section 2B and Supplementary Figure S4). The transition to the new energy state occurs infrequently and is accessed only from the conformational state associated with the more active-state-like dynamics of the receptor. This suggests that this new functional state that accompanies dimerization is associated with the active-state dimer. The second monomer (M2) of the dimer contains only one energy well (Fig. 6c). The motion of M2 within the energy basin displays localized motion in IL2 and at the C-terminus. The sampling of only a single energy state and the overall rigidity of the receptor suggests that M2 is somehow “turned-off” in the dimerized state. We have observed that in the rhodopsin dimers in general, when there is strong receptor-receptor interaction then only one of the monomers making up the dimer is functionally active and the other monomer is effectively “turned off”. Consequently, the weaker the interaction between the receptors forming the dimer – the more “monomer-like” or uncoupled are the internal dynamics of the individual monomers making up the dimer (Supplementary Information Section 3A and Supplementary Figures S4–S7).

In the P23H H1/H2/H8–H1/H2/H8 dimer both monomers making up the dimer are in a non-functional state (Fig. 6d,e). Each monomer has only a single energy well and analysis of the motion within each well features a highly deformed ligand-binding site with large-scale uncorrelated motion involving residues in the EC regions of helices H5 and H6 along with residues in EL2 and EL3. The deformation of the ligand-binding site, particularly in a region that strongly affects the dynamics of residues with an active-role in the retinal isomerization process would make the transition of the inactive monomers to the Meta-II state highly unlikely.

#### H4/H5–H4/H5 dimerization and modulation of P23H receptor function

**Figure 7:**
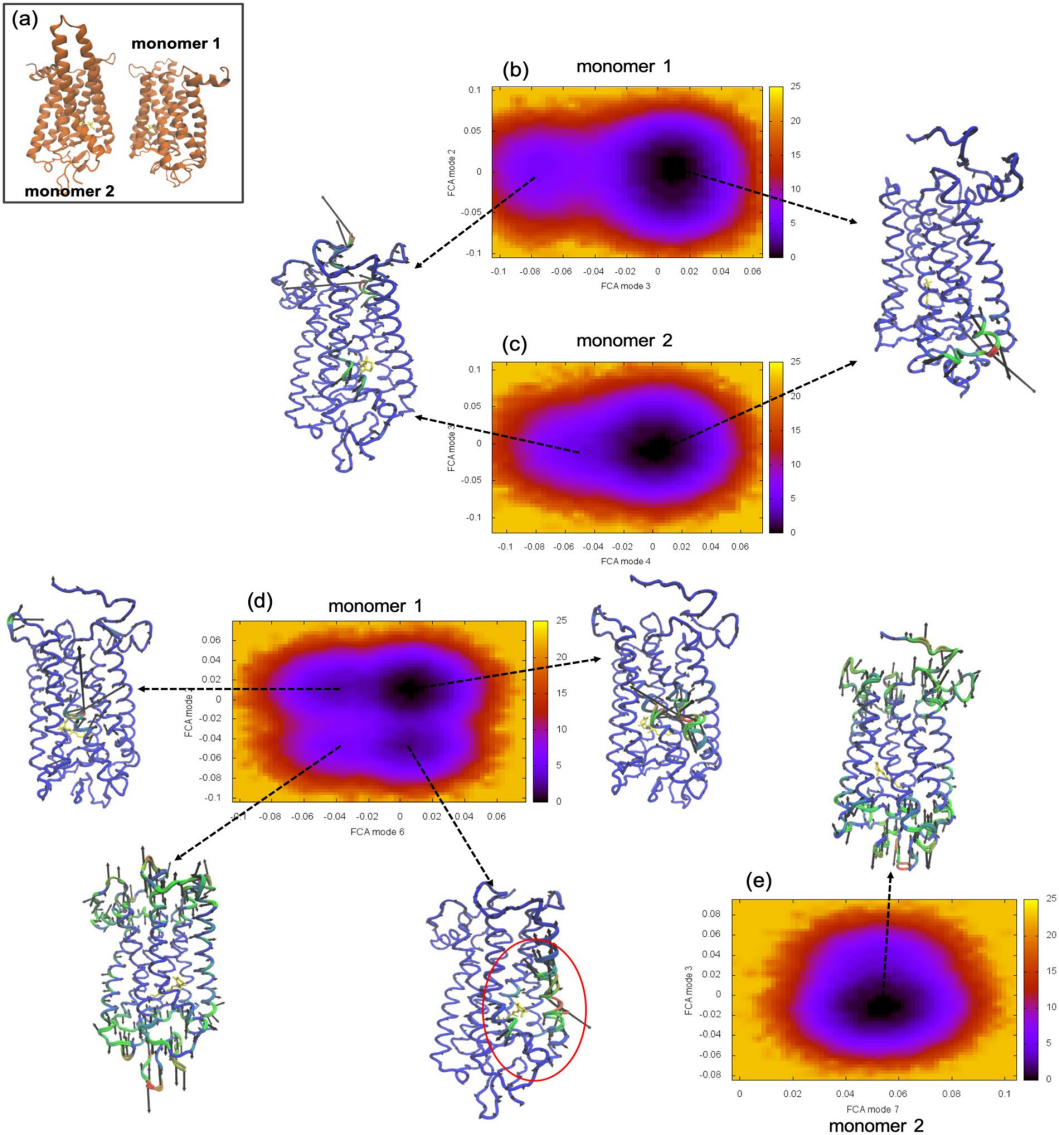
(**a**) Carton representation of WT rhodopsin molecules showing the arrangement of the receptors in the H4/H5–H4/H5 homodimer orientation. Free energy surfaces calculated from the FCA of the MD trajectories of (**b**) rhodopsin M1 and (**c**) M2 forming the WT dark-state H4/H5–H4/H5 dimer. The FCA of (**d**) M1 and (**e**) M2 from the P23H dark-state H4/H5–H4/H5 dimer MD simulations. The C−α representations of rhodopsin displays the principal motion of the receptor within the minimum of the energy wells.

Performing an analogous analysis on the H4/H5–H4/H5 dimer of WT rhodopsin in Fig. 7 it becomes apparent that there are weaker interactions between the individual monomers making up the dimer when contrasted with the H1/H2/H8–H1/H2/H8 dimer in Fig. 6. Both of the monomers (M1 and M2) of the dimer in Fig. 7b,c have a conformational energy landscape profile that more closely resembles the WT monomer (Fig. 3d), indicating that there is weaker coupling between the individual receptors. M1 (Fig. 7b) has a dual conformational profile. Again, the steeper basin is associated with the dark-state-like protein conformational fluctuations and the higher energy basin is associated with the active-state like dynamics. Although, in this case, the barrier separating the two states in M1 is considerably higher than the WT monomer - signifying also a higher barrier toward activation. The difference in barrier height is mainly attributed to the fact that the H4/H5–H4/H5 dimer distorts the retinal-binding region around helices H6 and H7, reducing the retinal-protein interactions in that region and as a consequence also weakening the transfer of energy from the retinal to the receptor during activation (Supplementary Figure S7). M2 (Fig. 7c) is dominated by dark-state-like conformational dynamics (the principal conformational energy state of the monomer) and has a dramatically reduced probability of transitioning to the active-state-like conformation when compared with either the WT monomer (Fig. 3d) or M1 of the H4/H5–H4/H5 dimer (Fig. 7b).

In the P23H H4/H5–H4/H5 dimer we witness two, new surprising features in the conformational landscape in Fig. 7d,e that differ from the P23H monomer in Fig. 3e and the P23H H1/H2/H8–H1/H2/H8 dimer in Fig. 6d,e. The first is the restoration of the sampling of dark-like and active-like-state conformational dynamics in M1 of the dimer that are comparable to the observed dynamics in the WT monomer (Fig. 3d). We have equated this ability to sample the inactive- and active-like conformational states in rhodopsin with receptor function. Secondly, we identify two new structures on the free energy surface that allude to an additional (secondary) functional state of the dimerized receptor that was not a part of the WT monomer population of conformations. We detect transitions from the dark-state-like conformation to a new conformation that involves an anticorrelated torsion that emanates from the retinal binding region (in the proximity of the β-ionone ring) and extends outward creating a substantial, intermittent cavity (Supplementary information, Section 2C and Supplementary Figure S5) between TM5 (Tyr206–Pro215) and TM6 (Ala269–Gly270). Additionally, we identify transitions from the cavity-forming torsional state to another new conformational state on the energy surface that involves a stretching-like torsion mainly localized at the receptor poles that is perhaps best described as a type of receptor axial elongation. The interactions between the two monomers forming the P23H H4/H5–H4/H5 dimer is strong, hence M2 has only a single conformational state on the free energy surface (“turned-off”). The primary dynamics taking place within the minima of M2 in Fig. 7e can be described as an axial elongation similar to the one identified from the free energy surface of M1 (Fig. 7d). In summary, we observe that this specific P23H dimer configuration forms a stable complex such that overall receptor function appears to be restored even though the Cys110–Cys187 disulfide bond remains ruptured in both monomers. Further, the original source of instability from mutation seems to also be the underlying basis for the emergence of an entirely new receptor function.

#### Meta-II-P23H H4/H5–H4/H5 homodimer

**Figure 8:**
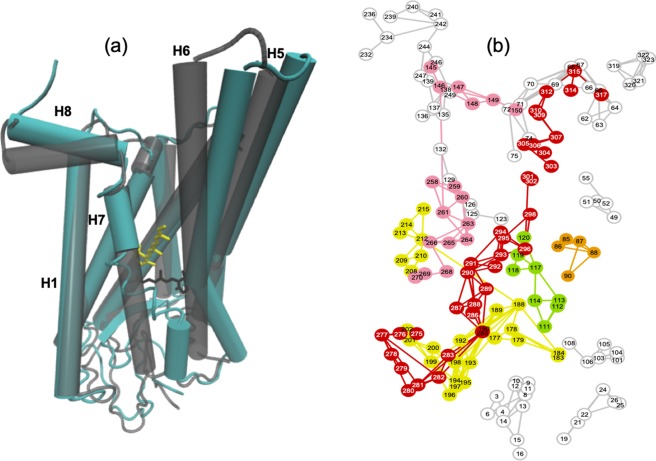
(**a**) Cartoon representation of monomer 1 of the H4/H5–H4/H5 Meta – II – P23H dimer (blue receptor and yellow retinal) overlaid with the structure of WT Meta-II (gray, transparent receptor and black retinal) from the MD simulations. (**b**) 2-D LSF showing the network of residue connections in M1 of the H4/H5–H4/H5 Meta-II-P23H dimer.

We have also performed MD simulations on the Meta-II-P23H H4/H5–H4/H5 dimer. Our aim is to gain insight into the nature of the new secondary function that emerged in the analysis of the inactive-state dimer. In all of the simulations performed, we find that the retinal of M1 is released from the receptor binding site. And this is coupled with the formation of a large cavity on the EC side of the ligand-binding region (Fig. 8a). In the active-state M1 we detect strong correlated dynamics between residues in helix H5 near the ligand-binding site with residues in EL2 (particularly Phe212 and Gly188) that are attributed to distortions from the inter-receptor residue interactions that stabilize the dimer (Fig. 8b). As a consequence, the EC side of helix H5 is distorted outward away from the transmembrane core. The dimer configuration also strongly correlates the motion of residues in helix H6 (Phe261) with residues in IL2 (Phe146) in M1. Therefore, the EC side of helix H6 is also strongly shifted outward away from the ligand-binding site. The EC changes of helices H5 and H6 are coupled with the outward pivot of the IC side of both helices, which subsequently destabilizes the highly conserved residue interactions between Tyr223 (H5) and Tyr306 (H7). The disruption of the conserved tyrosine residues on the IC side of the receptor permits the IC side of H7 to shift closer to H1 and the EC side of H7 to strongly shift closer to H6. Together, these changes create a large cavity at the EC side of M1 adjacent to helices H5–H7 and at the same time facilitates the release of the retinal from the ligand-binding site. M2 of the dimer has rather rigid dynamics (Supplementary Figure S9). The “turned-off” monomer (M2) has strongly correlated fluctuations between residues on H1 (Phe52) and H7 (Tyr301) that counter the induced dynamics caused by receptor association. The induced H1–H7 association also introduces slight structural changes within the ligand-binding site that directs the retinal to adopt an 11-*cis* configuration.

An obvious question that comes to mind is why this specific dimer configuration is correlated with the formation of a large cavity in the extracellular domain (ECD) of the active-receptor? One hypothesis is that the formation of the P23H homodimer is aligned with a specific ligand-based form of regulation and/or modulation of receptor function. Along these lines, it is interesting to note that we observe an enhancement of the signaling properties in the Meta-II-P23H H4/H5–H4/H5 homodimer when bound to the G-protein (Supplementary Figure S10). Explicitly, structural changes support a strengthened, continuous chain of intra-residue interactions that create an amplified signaling pathway extending from the extracellular side of the receptor through the microswitches on the intracellular side and up to the bound G-protein region. Although, in this configuration the G-protein bound Meta-II-P23H (H4/H5–H4/H5) dimer would presumably also be less sensitive to light when compared with the WT receptor. Specifically, the Meta-II-P23H dimer would produce a faster photo-response due to the structural modification in the ECD and in the IC regions around helices H6 and H7 and would be relatively inefficient due to the weakened interaction of the receptor with the retinal (Fig. 8, Supplementary Figure S13 and Supplementary information Section 4b).

## Discussion

In this investigation we have used both experimental and computational methods to investigate the functional dynamics and conformational stability of P23H rhodopsin. Our results are in agreement with previous investigations and demonstrate that P23H rhodopsin is thermally unstable and possesses an activation mechanism that differs significantly from that of the WT receptor. Our analyses suggest that the principal source of the functional differences is attributed to mutation-induced structural changes that lead to a ruptured disulfide bond (Cys110–Cys187) in the ECD and the consequent misfolding around the retinal-binding site that alters the stability of the Schiff base linkage - and hence also the pathway to activation. Our findings have also indicated that P23H is not functional as a monomer. Rather, the inherent instability of the receptor prompts P23H rhodopsin to adopt a specific homodimer arrangement: the P23H H4/H5–H4/H5 dimer. The P23H H4/H5–H4/H5 dimer reestablishes the principal function of the receptor and at the same time imparts a new, additional function. Incidentally, the structural changes that accompany dimerization in P23H Meta-II weaken the affinity of the receptor for the retinal while simultaneously supporting the formation of a hyper-functioning active-state. Interestingly, the P23H Meta-II dimer maintains a similar binding interface to the G-protein when compared to the WT receptor. This, coupled with the modified chromophore – receptor interaction denotes a probable altered intracellular signaling response in the P23H dimer^36^.

One approach for elucidating the context and significance of this emergent secondary function is to consider the possible physiological/functional significance of the observed preferential oligomeric state of the P23H rhodopsin mutant. For instance, there is accumulating evidence that strongly suggests that homodimerization is central in many aspects of GPCR biology – ranging from ontogeny to the regulation, formation and trafficking of GPCR-based signaling complexes. Particularly for rhodopsin, GPCR dimerization has been proposed to have a crucial role in the mechanism associated with biosynthesis and the subsequent export of the receptor from the endoplasmic reticulum (ER) to the cell surface^30,37,38^. A series of recent experimental measurements^37^ have confirmed that dimerization occurs early after biosynthesis in a number of other class A and class C GPCRs signifying that receptor dimerization may have a general role in receptor maturation.

Further, P23H rhodopsin is known to cause misfolding in the ER and as a result ER stress^39,40^. Cells subject to ER stress activate a network of signaling pathways referred to as the unfolded protein response or UPR to alleviate the stress, which if ineffective, leads to ER stress-associated programmed cell death or apoptosis. Previous studies on P23H rhodopsin have uncovered that in the early phase of UPR there is a marked increase in trafficking of the mutant receptor to the cell membrane^41^ and as a consequence ER apoptosis is suppressed. Rhodopsin uses the secretory pathway for intracellular trafficking^12,42,43^ indicating a direct connection between vesicular transport and ER homeostasis. Accordingly, it is now acknowledged that in some instances dimeric interfaces can provide an avenue for masking specific retention signals and/or hydrophobic patches of unfolded and misfolded mutant receptors^44,45^ that would normally be detected by the ER quality control system. This suggests that dimerization can, under certain conditions, functionally correct interactions or elicit conformational changes that are required to generate an appropriately folded receptor that is permitted to traffic to the cell surface.

There is also the changing view of P23H disease pathogenesis. It is now recognized that P23H rhodopsin accumulates in the rod outer segment (ROS) rather than the ER or Golgi as was initially thought^6,7,33,46^. The aggregates that form in the ROS are prone to form high molecular weight oligomeric structures that create abnormal internal membrane structures that are linked with progressive retinal degeneration. Even at very low concentrations, P23H has a destructive effect on disk membranes likely through a *homodimerization* process^6,33^. The mutant receptors self-aggregate but also aggressively associate with WT receptors^5^, disrupting the processing of normal opsin and inducing a toxic effect on healthy photoreceptors (Supplementary Information, Section 3c and Supplementary Figs. S14 and S15). Thus, the formation of the intracellular protein aggregates is principal to disease pathogenesis and postulates a direct mechanism for the progression of photoreceptor degradation.

## Conclusion

The results from this investigation indicate that the P23H mutation in rhodopsin represents a cellular-level allosteric perturbation. In other words, the local-level protein mutation has an altering effect on the cellular pathways that ultimately determine the function of the receptor. What are the connections that lead us to this conclusion? Allostery is when conformational perturbation at one location in a protein affects another location(s) at a distant site^47^. Consequently, perturbation at any site in the protein structure leads to a shift in the distribution of conformational states across the entire population of states^48,49^. Therefore, allosteric propagation enables communication between distinct sites in the protein structure and affects the equilibria of molecular interactions. In P23H rhodopsin we find that the perturbation due to mutation alters the distribution of conformational states. Experimentally, we detect these allosteric changes as an uncoupling of agonist dynamics with essential protein conformational changes that normally take place in the active-site WT receptor upon excitation. This uncoupling effect represents a mutation-induced allosteric perturbation on the local protein (molecular) level. Particularly, it signifies the modulation of a major allosteric propagation pathway in rhodopsin that disrupts the intra-protein communication that connects the ligand-binding site with the G-protein signaling region. Investigating further, we discover that this protein-level perturbation may have ramifications on the network of downstream events that shape the cellular response of the receptor as a whole. To understand this connection, it is important to recognize that signaling pathways are comprised of a series of molecular interactions. And cellular networks consist of a succession of interconnected signaling pathways. Thus, changing the distribution of conformational ensembles of the individual proteins comprising the signaling pathways has an overall effect on the interconnectivity and outcome of the cellular response^48,50^.

In P23H rhodopsin, we find that mutation shifts the ensemble toward a conformation that increases the probability of dimer formation, while diminishing the importance of retinal-binding as a prerequisite for receptor function. Thus the initial protein-level allosteric perturbation could plausibly modify the global allosteric effects that collectively communicate and translate incoming signals to cell actions. In this interpretation, the modulation of the network of pathways defining rod *cellular* function foster the formation of a receptor with indistinct ligand specificity and a distinct new function. Specifically, we observe that changes in ligand-binding that accompany homodimerization give rise to a functional active-state receptor that can effectively couple to the G-protein but likely with an altered signaling cascade.

Overall, our findings suggest that it may be necessary to revise how one views P23H-linked ADRP and to reconsider the therapeutic approaches to treat it. Currently, most approaches have focused on therapies that emphasize mechanisms that act on the single protein-level for diminishing/alleviating the effects of RP^51–54^. However, these types of approaches have to date met with limited success. Our results indicate that an approach that incorporates allosteric strategies that target or track specific cellular signaling pathways, such as those currently being considered for a number of aggressive types of cancer^55–57^, may offer a fresh perspective on the development of therapeutic potentials.

## Materials and Methods

Single amino acid replacements P23H was prepared by a two-step PCR mutagenesis technique using the synthetic bovine opsin gene in the expression vector pMT4^58^, followed by sub-cloning of the mutant fragment using unique KpnI and NotI restriction sites into the tetracycline inducible expression system vector pACMV-tetO^59^, as described in detail (Mitchell *et al*., 2019). The tetracycline inducible HEK293S stable cell lines expressing P23H rhodopsin was established as described based on previously published protocols^59^. Details of other methods, including spectroscopy measurements and molecular dynamics (MD) simulation protocols and analyses are presented in Supplementary information, *Materials and Methods*.

## Supplementary information

Supplementary Data.

## References

[1] Illing ME, Rajan RS, Bence NF, Kopito RR (2002). A Rhodopsin Mutant Linked to Autosomal Dominant Retinitis Pigmentosa Is Prone to Aggregate and Interacts with the Ubiquitin Proteasome System. J. Biol. Chem..

[2] Athanasiou D (2018). The molecular and cellular basis of rhodopsin retinitis pigmentosa reveals potential strategies for therapy. Prog. Retin. Eye Res..

[3] Kaushal S, Khorana HG (1994). Structure and Function in Rhodopsin. 7. Point Mutations Associated with Autosomal Dominant Retinitis Pigmentosa. Biochemistry.

[4] Chen Y (2014). Inherent instability of the retinitis pigmentosa P23H mutant opsin. J. Biol. Chem..

[5] Rajan RS, Kopito RR (2005). Suppression of wild-type rhodopsin maturation by mutants linked to autosomal dominant retinitis pigmentosa. J. Biol. Chem..

[6] Haeri M, Knox BE (2012). Rhodopsin Mutant P23H Destabilizes Rod Photoreceptor Disk Membranes. PLOS ONE.

[7] Sakami S (2011). Probing mechanisms of photoreceptor degeneration in a new mouse model of the common form of autosomal dominant retinitis pigmentosa due to P23H opsin mutations. J. Biol. Chem..

[8] Behnen P (2018). A Small Chaperone Improves Folding and Routing of Rhodopsin Mutants Linked to Inherited Blindness. iScience.

[9] Frauenfelder H, Sligar S, Wolynes P (1991). The energy landscapes and motions of proteins. Science.

[10] Carrell RW, Lomas DA (1997). Conformational disease. The Lancet.

[11] Woods KN, Pfeffer J, Dutta A, Klein-Seetharaman J (2016). Vibrational resonance, allostery, and activation in rhodopsin-like G protein-coupled receptors. Sci. Rep..

[12] Noorwez SM (2004). Retinoids Assist the Cellular Folding of the Autosomal Dominant Retinitis Pigmentosa Opsin Mutant P23H. J. Biol. Chem..

[13] Woods KN (2010). Solvent-induced backbone fluctuations and the collective librational dynamics of lysozyme studied by terahertz spectroscopy. Phys. Rev. E.

[14] Woods KN (2014). The glassy state of crambin and the THz time scale protein-solvent fluctuations possibly related to protein function. BMC Biophys..

[15] Woods, K. N., Pfeffer, J. & Klein-Seetharaman, J. Chlorophyll-Derivative Modulation of Rhodopsin Signaling Properties through Evolutionarily Conserved Interaction Pathways. *Front. Mol. Biosci*. **4**, (2017). 10.3389/fmolb.2017.00085 PMC5733091 29312953

[16] Jardón-Valadez E, Bondar A-N, Tobias DJ (2010). Coupling of Retinal, Protein, and Water Dynamics in Squid Rhodopsin. Biophys. J..

[17] Kimata N (2016). Retinal orientation and interactions in rhodopsin reveal a two-stage trigger mechanism for activation. Nat. Commun..

[18] Schnedermann C, Liebel M, Kukura P (2015). Mode-Specificity of Vibrationally Coherent Internal Conversion in Rhodopsin during the Primary Visual Event. J. Am. Chem. Soc..

[19] Johnson PJM (2015). Local vibrational coherences drive the primary photochemistry of vision. Nat. Chem..

[20] Ramanathan A, Savol A, Burger V, Chennubhotla CS, Agarwal PK (2014). Protein Conformational Populations and Functionally Relevant Substates. Acc. Chem. Res..

[21] Ramanathan A, Savol AJ, Langmead CJ, Agarwal PK, Chennubhotla CS (2011). Discovering Conformational Sub-States Relevant to Protein Function. PLOS ONE.

[22] Ma B, Nussinov R (2016). Protein dynamics: Conformational footprints. Nat. Chem. Biol..

[23] Ishikawa H, Kwak K, Chung JK, Kim S, Fayer MD (2008). Direct observation of fast protein conformational switching. Proc. Natl. Acad. Sci..

[24] Frauenfelder H, Parak F, Young RD (1988). Conformational Substates in Proteins. Annu. Rev. Biophys. Biophys. Chem..

[25] Mitchell J (2019). Comparison of the molecular properties of retinitis pigmentosa P23H and N15S amino acid replacements in rhodopsin. PLOS ONE.

[26] Zhou XE, Melcher K, Xu HE (2012). Structure and activation of rhodopsin. Acta Pharmacol. Sin..

[27] Knepp AM, Periole X, Marrink SJ, Sakmar TP, Huber T (2012). Rhodopsin forms a dimer with cytoplasmic helix 8 contacts in native membranes. Biochemistry.

[28] Schertler GF, Hargrave PA (1995). Projection structure of frog rhodopsin in two crystal forms. Proc. Natl. Acad. Sci..

[29] Miller LM, Gragg M, Kim TG, Park PS–H (2015). Misfolded Opsin Mutants Display Elevated β-Sheet Structure. FEBS Lett..

[30] Terrillon S, Bouvier M (2004). Roles of G-protein-coupled receptor dimerization. EMBO Rep..

[31] Devi LA (2001). Heterodimerization of G-protein-coupled receptors: pharmacology, signaling and trafficking. Trends Pharmacol. Sci..

[32] Gurevich VV, Gurevich EV (2008). How and why do GPCRs dimerize?. Trends Pharmacol. Sci..

[33] Saliba RS, Munro PMG, Luthert PJ, Cheetham ME (2002). The cellular fate of mutant rhodopsin: quality control, degradation and aggresome formation. J. Cell Sci..

[34] Tam BM, Moritz OL (2006). Characterization of Rhodopsin P23H-Induced Retinal Degeneration in a Xenopus laevis Model of Retinitis Pigmentosa. Invest. Ophthalmol. Vis. Sci..

[35] Periole X, Knepp AM, Sakmar TP, Marrink SJ, Huber T (2012). Structural Determinants of the Supramolecular Organization of G Protein-Coupled Receptors in Bilayers. J. Am. Chem. Soc..

[36] Weis WI, Kobilka BK (2018). The Molecular Basis of G Protein–Coupled Receptor Activation. Annu. Rev. Biochem..

[37] Bulenger S, Marullo S, Bouvier M (2005). Emerging role of homo- and heterodimerization in G-protein-coupled receptor biosynthesis and maturation. Trends Pharmacol. Sci..

[38] Milligan G (2010). The role of dimerisation in the cellular trafficking of G-protein-coupled receptors. Curr. Opin. Pharmacol..

[39] Athanasiou D (2013). The cell stress machinery and retinal degeneration. FEBS Lett..

[40] Athanasiou D (2017). The role of the ER stress-response protein PERK in rhodopsin retinitis pigmentosa. Hum. Mol. Genet..

[41] Gorbatyuk M. S., Knox T., LaVail M. M., Gorbatyuk O. S., Noorwez S. M., Hauswirth W. W., Lin J. H., Muzyczka N., Lewin A. S. (2010). Restoration of visual function in P23H rhodopsin transgenic rats by gene delivery of BiP/Grp78. Proceedings of the National Academy of Sciences.

[42] Deretic D (1998). Post-Golgi trafficking of rhodopsin in retinal photoreceptors. Eye.

[43] Satoh AK, O’Tousa JE, Ozaki K, Ready DF (2005). Rab11 mediates post-Golgi trafficking of rhodopsin to the photosensitive apical membrane of Drosophila photoreceptors. Development.

[44] Margeta-Mitrovic M, Jan YN, Jan LY (2000). A Trafficking Checkpoint Controls GABAB Receptor Heterodimerization. Neuron.

[45] Kobayashi H, Ogawa K, Yao R, Lichtarge O, Bouvier M (2009). Functional Rescue of β1-Adrenoceptor Dimerization and Trafficking by Pharmacological Chaperones. Traffic Cph. Den..

[46] Sakami S, Kolesnikov AV, Kefalov VJ, Palczewski K (2014). P23H opsin knock-in mice reveal a novel step in retinal rod disc morphogenesis. Hum. Mol. Genet..

[47] Guo J, Zhou H-X (2016). Protein Allostery and Conformational Dynamics. Chem. Rev..

[48] Nussinov R, Tsai C-J, Liu J (2014). Principles of Allosteric Interactions in Cell Signaling. J. Am. Chem. Soc..

[49] Fenwick RB, Esteban-Martín S, Salvatella X (2011). Understanding biomolecular motion, recognition, and allostery by use of conformational ensembles. Eur. Biophys. J..

[50] Nussinov R, Tsai C-J (2013). Allostery in Disease and in Drug Discovery. Cell..

[51] Mendes HF, Cheetham ME (2008). Pharmacological manipulation of gain-of-function and dominant-negative mechanisms in rhodopsin retinitis pigmentosa. Hum. Mol. Genet..

[52] Vasireddy V (2011). Rescue of Photoreceptor Degeneration by Curcumin in Transgenic Rats with P23H Rhodopsin Mutation. PLOS ONE.

[53] Tam BM, Moritz OL (2007). Dark Rearing Rescues P23H Rhodopsin-Induced Retinal Degeneration in a Transgenic Xenopus laevis Model of Retinitis Pigmentosa: A Chromophore-Dependent Mechanism Characterized by Production of N-Terminally Truncated Mutant Rhodopsin. J. Neurosci..

[54] Price BA (2011). Mislocalization and Degradation of Human P23H-Rhodopsin-GFP in a Knockin Mouse Model of Retinitis Pigmentosa. Invest. Ophthalmol. Vis. Sci..

[55] Downward J (2003). Targeting RAS signalling pathways in cancer therapy. Nat. Rev. Cancer.

[56] Bild AH (2006). Oncogenic pathway signatures in human cancers as a guide to targeted therapies. Nature.

[57] Zhu P, Aliabadi HM, Uludağ H, Han J (2016). Identification of Potential Drug Targets in Cancer Signaling Pathways using Stochastic Logical Models. Sci. Rep..

[58] Oprian DD, Molday RS, Kaufman RJ, Khorana HG (1987). Expression of a synthetic bovine rhodopsin gene in monkey kidney cells. Proc. Natl. Acad. Sci. USA.

[59] Reeves PJ, Kim J-M, Khorana HG (2002). Structure and function in rhodopsin: a tetracycline-inducible system in stable mammalian cell lines for high-level expression of opsin mutants. Proc. Natl. Acad. Sci. USA.

