# Supplementary material for: Conformational perturbation, allosteric modulation of cellular signaling pathways, and disease in P23H rhodopsin
Source: Sci Rep. 2020 Feb 14;10:2657. doi: 10.1038/s41598-020-59583-2 (PMC7021821; doi:10.1038/s41598-020-59583-2)
Supplement: Supplementary file 1 — Supplementary Data. [file 41598_2020_59583_MOESM1_ESM.pdf]

Conformational perturbation, allosteric modulation of cellular signaling pathways, and disease in P23H rhodopsin

Kristina N. Woods[1*] and Jürgen Pfeffer[2]

[1] *Lehrstuhl für BioMolekulare Optik, Ludwig-Maximilians-Universität, 80538 München, Germany*

[2] *Technical University of Munich, Bavarian School of Public Policy, 80333 München, Germany*

Supplementary information

* Corresponding author: kristina.woods@physik.lmu.de

Materials and Methods

Rhodopsin and P23H rhodopsin sample preparation

Materials

1D4 antibody was obtained from Dr. Robert Molday of the University of British Columbia, Vancouver, BC, Canada. 9-*cis* retinal was purchased from Sigma. Dodecyl maltoside was purchased from Anatrace.

Expression

Single amino acid replacements P23H was prepared by a two-step PCR mutagenesis technique using the synthetic bovine opsin gene in the expression vector pMT4(1), followed by sub-cloning of the mutant fragment using unique KpnI and NotI restriction sites into the tetracycline inducible expression system vector pACMV-tetO(2), as described in detail (see supplement, Mitchell et al., 2019). The tetracycline inducible HEK293S stable cell lines expressing P23H rhodopsin was established as described (Mitchell et al., 2019) based on previously published protocols(2). HEK293S cells stably expressing P23H rhodopsin were grown in complete media and when cells were about 80-90% confluent, they were washed twice with phosphate buffered saline (PBS) and induced with induction media: complete media with tetracycline (2µg/ml) and sodium butyrate (5mM). At 2 and 24 hours after induction, *9-cis* retinal (chromophore) was added directly to the cells at 10µM concentration so that the final concentration of the chromophore is 20µM(3). The chromophore was added in the dark and all subsequent steps were also performed in the dark. Cells were harvested 48 hours after induction.

Protein purification

Proteins were solubilized in DM and purified by ID4 immunoaffinity chromatography in 0.05% DM as described(4, 5). Solubilized cells were centrifuged for 30 min at 35,000 rpm and 4^0C. The supernatant was mixed with 1D4 Sepharose beads (approximate binding capacity of 1 µg rhodopsin/ µl of resin) for at least 6 h at 4^0C. The resin was then washed with 50 bed volumes of 0.05% (w/v) DM in PBS followed by 10 bed volumes of 0.05% (w/v) DM in 2 mM Na_2HPO_4/NaH_2PO_4 (pH 6.0). WT, P23H and N15S proteins were eluted with 70µM C-terminal nonapeptide (TETSQVAPA) in 0.05% (w/v) DM in 2 mM Na_2HPO_4/NaH_2PO_4 (pH 6.0).

TeraHertz (THz) spectroscopy experiment

The dark-state rhodopsin and P23H rhodopsin experiments were performed under dim-red light conditions. Subsequent photo-isomerization was triggered with visual light excitation. The THz spectroscopy experiments were carried out on a Jasco FTIR - 6000 series spectrometer. The rhodopsin sample spectra were collected with a liquid helium cooled bolometer in the 15–250 cm^{-1} spectral range. The 15-100 cm^{-1} THz spectra were collected with a 25-micron beam splitter while the data in the 100–250 cm^{-1} spectral region was collected with a 12-micron beam splitter. For each transmission measurement a 25 mm diameter region of the sample was illuminated with the THz beam to determine the absorbance. In the spectral measurements presented, each scan consists of 16 averaged scans and the infrared data was collected with a spectral resolution of 4 cm^{-1}.

Computation

Molecular dynamics (MD) simulations of GPCRs

MD Simulation of rhodopsin and P23H rhodopsin

Each MD simulation consisted of a starting x-ray crystal structure taken from the PDB database. PDB structure 1u19 was used for the inactive (dark) state of rhodopsin and 3pxo was used for Meta II. In all simulations, the receptor was embedded in a hydrated lipid bilayer with all atoms represented explicitly. The CHARMM-GUI membrane builder was used for building the protein-membrane complex for all MD simulations. Specifically, the receptor and any resolved water molecules from the crystal structure were used as starting structures to embed in an equilibrated palmitoyloleoyl-phosphatidylcholine (POPC) bilayer. Subsequently, water molecules and NaCl ions were added to hydrate and neutralize the net charge of the system. In the rhodopsin simulations, all titratable groups in the receptor were considered to be charged (6). The exceptions were Asp83 and Glu122, which were both neutral in both the dark-state and Meta II MD simulations. For the dark-state rhodopsin MD simulations, the Schiff base was protonated whereas Glu113 was deprotonated. For the Meta-II simulations both the Schiff base and Glu113 were set to neutral.

MD simulations were performed at 300K using the Gromacs package (www.gromacs.org) version 5.0. The all-atom CHARMM36m force field parameters were utilized for the protein and for the lipid parameters. The SPC water model was used for hydration and the ground-state retinal parameters (7) for both the 11-*cis* and *all-trans* retinal chromophore were obtained from the Bondar group.

In cases where the receptor structures contained thermostabilizing mutations, they were mutated back to the wildtype sequence. Regions with missing residues, particularly of the third intracellular loop, were added to the protein structure by using the homology modelling program MODELLER (https://salilab.org/modeller/). In all MD simulations carried out in this work energy minimizations were initially carried out to reduce the number of unfavorable contacts between added solvent molecules, lipids and the receptor by using a steepest descent method to a convergence tolerance of 0.001 kJ mol^{-1}. The energy minimization was followed by a MD run with constraints for 200 ps in which an isotropic force constant of 100 kJ mol^{-1} nm^{-1} was used on the protein and lipid atoms. During the restrained dynamics simulation, the temperature and pressure of the system were kept constant by weak coupling to a modified velocity rescaled Berendsen temperature (8) and pressure baths and in all cases the protein, lipid, water, and ions were coupled to the temperature and pressure baths separately. The output conformation from the MD simulation with constraints was used as the starting conformation for an initial 300 ns equilibrium MD simulation.

Ten subsequent simulations were conducted where randomized conformations from the last 10 ns of the equilibrium simulations were used as starting point conformations for each distinct simulation. These subsequent simulations were carried out for an additional 800 ns and were eventually used to assess the picosecond time scale fluctuations in the receptor systems. The final simulations were carried out with a 2 fs time step where the bonds between the hydrogen and the other heavier atoms were restrained to their equilibrium values with the linear constraints (LINCS) algorithm (9). Particle mesh Ewald (PME) method (10) was used to calculate the long-

range electrostatic interactions in the simulation and was used with a real-space cutoff of 1.0 nm, a fourth order B-spline interpolation and a minimum grid spacing of 0.14 nm.

MD simulations of rhodopsin P23H mutant were carried out by creating a residue mutation with DUET (http://biosig.unimelb.edu.au/duet/stability), a web server for studying missense mutations in proteins. Similarly, back mutations to restore thermostabilizing mutations in the crystal structures of WT receptors to their wildtype sequence were also carried out with DUET. Minimization and production run MD simulations on the mutated and back mutated receptors were carried out in a manner analogous to that described for the WT receptors.

Coarse-grained self-assembly MD simulations of rhodopsin and P23H rhodopsin
Coarse-Grained Molecular Dynamics (CG MD) simulations to determine the self-assembly characteristics of rhodopsin and P23H rhodopsin were performed using the MARTINI Coarse-Grained force field and its extension to proteins (http://cgmartini.nl/). The self-assembly simulations consisted of 16 lipid bilayer embedded rhodopsin or P23H rhodopsin molecules in a protein-to-lipid ratio of 1/100. Both the CG model for rhodopsin and P23H rhodopsin were based on the output structures from our initial MD simulations on both receptors described in a previous section, although the retinal molecule was not included in the models. Initially, an atomistic model of the individual rhodopsin molecules were placed on a 4x4 grid that positioned them equidistant from one another. The rhodopsin system of molecules was subsequently embedded into a pre-equilibrated POPC lipid bilayer and the entire system was then translated into a Martini protein and lipid topology with a Martini python script. All CG MD simulations were carried out with Gromacs 5.0. The Martini v2 forcefield parameters were used for protein interactions and the Martini POPC topology for lipids. Ten distinct simulations on the system of rhodopsin molecules were carried out and were set up by initializing a random seed generator that was applied to positional restraints on the central backbone bead of Gly121 of each receptor in the original array. In this way, the relative orientation of the individual receptors were randomized while maintaining their initial spatial distribution in the lipid bilayer. The system of rhodopsin molecules were run for 100 microseconds with a 20 fs timestep. In the simulations, Coulomb and van der Waal potentials had a cut-off of 1.2 nm and both the temperature and pressure were weakly coupled to a Berendsen thermostat and maintained at 300 K and 1 atm, respectively.

Cluster analyses - as implemented in GROMACS - were used to resolve the receptor dimer pairs formed from the self-aggregation simulations. The matrix of positional root-mean-square-difference (RMSD) of the backbone beads of the receptor dimers with a cut-off of 0.4 nm was used as an input for the cluster analysis.

The dimers identified from the CG MD simulations were converted from a coarse-grained representation back to an all-atom representation for atomistic MD simulations. Using a structural model of a rhodopsin monomer, retinal molecules were placed back in the rhodopsin dimer structures and the dimer was embedded in a pre-equilibrated POPC bilayer. MD simulations were conducted on the all-atom representation of the rhodopsin dimers in a manner analogous to that outlined for rhodopsin monomers in a previous section.

Umbrella sampling for calculation of the potentials of mean force (PMFs)
Umbrella sampling was used to obtain the PMFs and subsequently the energy of dimer association interfaces of the identified dimers from the CG simulations. The umbrella sampling was carried out by using simulation windows in which one monomer (of the dimer) was restrained and the other (monomer) pulled away in order to generate a series of configurations along the reaction coordinate. The reaction coordinate in our calculation was the y – axis and the configurations were extracted from the atomistic simulations carried out on the identified dimers.

For each configuration, umbrella sampling windows were distributed at positions that corresponded to varying inter-protein separation distances along a line connecting the center of mass of the two monomers. The umbrella potential was applied to the center of mass of each of the monomer's C_{α}- atoms. The sampling windows were distributed at 0.1 nm interval starting from a protein inter-separation distance of 2.8 nm up to 4 nm and a spacing of 0.2 nm was used up to 8 nm. Each of the simulation windows had a harmonic umbrella potential with a force constant of 1000 kJ mol^{-1} nm^{-1}. A converged calculation of the PMF requires efficient sampling of configurations along the reaction coordinate so that there is an overlap between successive histograms from adjacent simulation windows (i.e. the histograms are smooth). The weighed histogram analysis method (WHAM) was used to calculate the PMF from the individual configurations along the reaction coordinate to determine the association interfaces of the rhodopsin homodimers.

MD simulation of Meta-II and Meta-II-P23H in complex with the G-protein Transducin (G_s)
The crystal structure of the G-protein transducin (G_S) in complex with the β2 - adrenergic receptor (3sn6) along with crystal structure of the active G-protein-coupled receptor opsin in complex with a C-terminal peptide derived from the G-alpha subunit of transducin (3dqb) were used as starting structures for aligning G_s onto the Meta-II and Meta-II-P23H structures. The G-protein complexed receptors were embedded into a membrane environment in an equivalent manner as described above for the non-complexed receptors and minimization and equilibration simulations were carried out before performing production runs for a total of 1.2 μs.

An equivalent method was used for aligning and carrying out the G-protein simulations in the Meta-II and Meta-II-P23H *dimers*.

Principal component analysis (PCA*)*
Principal component analysis or PCA is generally employed to detect correlations in large data sets. In MD simulations, the method can be utilized to reveal the most important motions in proteins. In this study, principal component analyses (PCAs) were carried out by diagonalzing the covariance matrix $C_{ij=\langle(x_i-\langle x_i\rangle)(x_j-\langle x_j\rangle)\rangle}$, where x denotes protein atomic positions in the $3N$-dimensional conformational space and the angular brackets represent the averages over the MD trajectory. Translational and rotational motions were removed by a least squares fitting to a reference structure. The eigenvectors of $\boldsymbol{C}$ were determined by diagonalization with an orthonormal transformation matrix. The resulting eigenvectors from the transformation were used to determine the PCA modes with eigenvalues (λ) equivalent to the variance in the direction of the corresponding eigenvector. The MD trajectory was projected onto the principal modes to determine the principal components. The eigenvalues λ_i of the principal components

denote the mean square fluctuation of the principal component i and are arranged so that $\lambda_1 \geq \lambda_2 \geq \cdots \geq \lambda_{3N}$. Using this arrangement, the trajectories were filtered along the first principal component to analyze the collective dynamics taking place within the protein. The cosine content of the PCA modes presented were found to be less than 0.001.

Full Correlation Analysis (FCA)
The full correlation analysis (FCA) algorithm (11) based on mutual information (MI) was used to detect correlated motions of the proteins from the MD simulations. The g_fca tool used in GROMACS utilizes a previously calculated PCA as a starting point for the optimization. FCA minimizes the coupling between the coordinates and the FCA modes are ranked by anharmonicity (rather than fluctuation amplitude). In this case, the anharmonicity of a FCA mode is defined by the difference in the information-entropy of the observed density and that of a Gaussian function with the same variance. The visualization of the essential FCA modes and a description of the dynamics within the protein conformational subspace were conducted by selecting the FCA modes with the highest pairwise correlation. The free energy surface plots of the selected pair of FCA modes were created with the gmx sham tool in GROMACS and further visualized with gnuplot.

Localized structural fluctuations (LSFs)
Localized structural fluctuations (LSFs) are local relaxations that reflect specific intramolecular and intermolecular induced protein fluctuations. LSFs have also been hypothesized to form the basis of allosteric signal propagation in proteins (12). The localized structural fluctuations from the MD simulations carried out on rhodopsin were calculated with the method of Pandini *et al.*(13) that utilizes a structural alphabet (SA) to define protein local structural fluctuations that are described by a set of 25 canonical states composed of four-residue protein fragments. The four-residue fragments define the most probable protein local, conformational fluctuations in the protein 3-D structure. Structural correlations between local conformational changes of two protein fragments were calculated as a positional mutual information (MI) matrix between two column positions in the SA alignment.

Calculation of pair-wise forces from MD simulation
A modified version of Gromacs 4.5.3 was used to write out the pair-wise forces(14), Fij, between each residue pair i and j. Forces include contributions from the electrostatic and van der Waals interactions involving the G-protein and rhodopsin (Meta-II) or rhodopsin and the retinal that are calculated below a certain cut-off distance. These non-bonded pairwise forces of the residue pairs in close proximity comprise a force-propagation network involving short-range to medium-range connections that are averaged over the simulation time. The averaged forces were saved every 10 ps and convergence was reached when an equilibrium value for the forces was attained. The stored forces were written out as force trajectories and the average of those forces were used later for further analysis in R (https://www.r-project.org/) as well as visualization in VMD (http://www.ks.uiuc.edu/Research/vmd/). Covariance matrices and principal component analyses (PCA) were used on the averaged residue forces to identify correlated changes in the pair-wise forces between rhodopsin and the G-protein transducin in both Meta-II structures.

Visualization of protein networks

LSF networks were created with the iGraph package in R. Connected components with four or less residues were removed to increase readability.

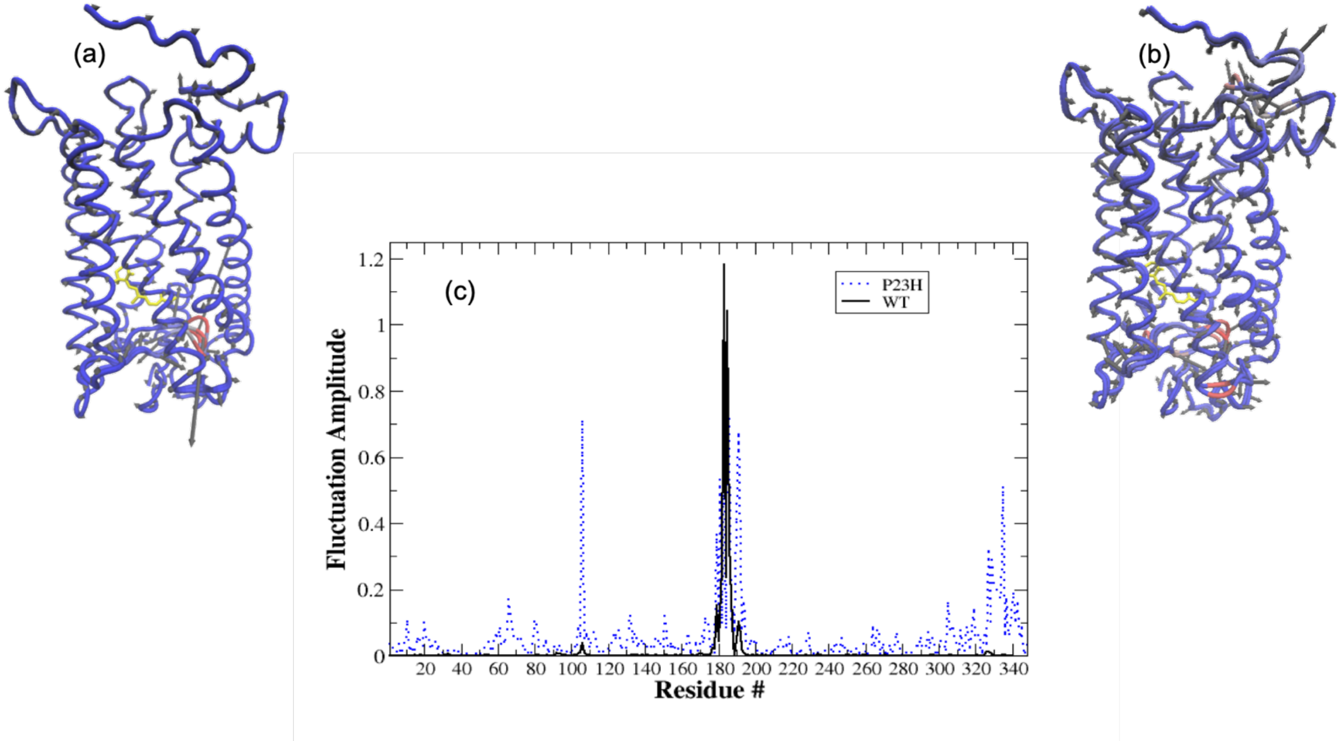

Supplementary Figure S1: : C-α representation of the dominant PCA mode (PCA1) from the MD simulation of the dark-state **(a)** WT rhodopsin and **(b)** P23H rhodopsin where the areas in blue show regions with less mobility and the areas in red show regions with more mobility and (**c**) the corresponding per residue fluctuation amplitude of WT (black, solid line) and P23H (blue, dotted line) rhodopsin .

Section 1: Force analysis of rhodopsin-retinal interactions in dark-state P23H and WT rhodopsin

A closer look at the nature of the receptor-retinal coupling interactions in the dark-state of P23H rhodopsin provides further insight into the molecular mechanisms that link the single-point mutation in the N-terminus with the overall biochemical instability of the receptor. For instance from analyzing the two dominant conformations that arise from retinal-rhodopsin weak force interactions from the MD simulation we find that the disrupted network of H-bonding interactions near the PSB also displaces conserved water molecules that mediate the activation signals from the retinal-binding pocket to the cytoplasmic interface of rhodopsin. For instance, in Supplementary Figure S2a we determine that the displacement of the structural water molecules in the retinal ligand-binding region is coupled with large-amplitude fluctuations of EL2 and EL3 that modulate the packing interactions in the receptor hydrophobic core as well as the H-bond network of interactions surrounding the retinal. Specifically, the correlated oscillations of side-chains of Glu181 and Ser186 – Tyr191 in EL2 along with Pro285 in EL3 are coupled with prominent side-chain fluctuations of Leu119-Glu122 in helix H3. These coupled set of fluctuations alter the stabilizing network of H-bonds in the ligand-binding pocket and modify the electrostatic properties on both the polyene chain and retinal ring, both of which are associated with the overall thermal stability of the receptor. The displacement of water molecules in the ligand-binding pocket is also connected with large amplitude fluctuations of residues associated with the shift of the counterion during activation. In Supplementary Figure S2b we observe that the other dominant conformation in the dark-state receptor involves large-

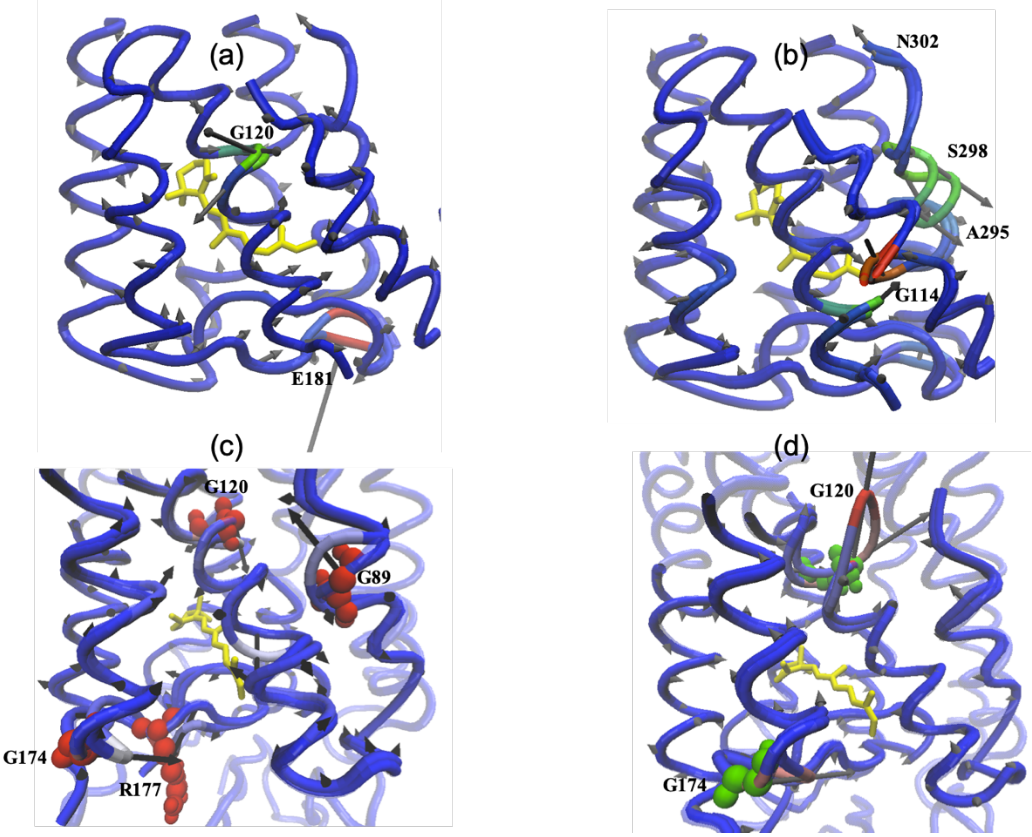

Supplementary Figure S2: The dominant modes of fluctuation derived from a PCA of the force trajectory of P23H dark-state rhodopsin is mapped onto a C-α representation of the receptor. The arrows show the direction and amplitude of the extreme residue positions when projecting along the **(a)** first eigenvector and **(b)** the second eigenvector. The analogous analysis has been done on the WT dark-state receptor in (**c**) and (**d**). The C–α representation of the receptor is color coded to reflect the amplitudes of the induced fluctuations. Regions in blue are less mobile and regions in red are more mobile.

amplitude oscillations of Glu113 coupled with fluctuations of Gly89 and Gly90 on helix H2 that arise from the reduction of H-bonds in the network of interactions stabilizing the Schiff base. The correlated fluctuations move Glu113 away from the PSB, which consequently modifies the electrostatic interactions in the salt bridge between the retinal and Schiff base. The motion of the counterion for the protonated Schiff base (Glu113) is also directly linked, through the same H-bonding network of interactions, with the increased conformational flexibility of residues that form the conserved NPxxY motif in rhodopsin and other Class-A GPCRs. Supplementary Figure S2b shows that out-of-phase correlated fluctuations link the prominent oscillations of the Glu113 Schiff base network with the increased torsional fluctuations of residues Ala295 – Asn302 in helix H7.

The analyses of the receptor-chromophore interactions in P23H dark-state rhodopsin indicate that the mutation affects an extensive network of interhelical interactions that stabilize the ground state of the receptor. The disruption of this network creates electrostatic changes around the ligand-binding pocket that lowers the energy gap between the ground and excited of the retinal, and hence has an overall effect on the thermal stability of the entire receptor. Furthermore, the modifications in these networks of interactions surrounding the retinal ligand-binding pocket reveal the underlying role of conformational plasticity in the biochemical

instability of the mutated receptor. The biochemical effect is two-fold: disruption of the stabilizing interactions in the Schiff base H-bonding network would negatively affect the probability that the shift in counterion to activate the G-protein would occur during receptor activation. Analogously, the amplified dynamics in the conserved microdomain region would create a physical disconnect in the receptor 3-D structure that would hinder the coupling of discrete components of conformational fluctuations that are required for modulating the excitation signal from the active-site to the intracellular side of the receptor where activation occurs.

The P23H receptor dynamics is in stark contrast to the observed retinal-rhodopsin weak interactions in the WT receptor in Supplementary Figures S2c – d. The force from the retinal in the dark-state in the WT receptor is distributed more extensively on the receptor structure. From the MD simulations, we are able to establish a network of interactions that effectively propagate internal strain from the ligand-binding site to the rest of the receptor structure via subtle, structural fluctuations. In the calculation of the pair-wise forces in the dark-state, we find that the retinal interaction with the receptor promotes two distinct mechanisms of force propagation that lead to conformational rearrangements within the receptor interior. The major pathway in Supplementary Figure S2c involves a torsional compression of the retinal-ligand binding pocket that is primarily due to the force on residues Glu113 and Gly114 from the oscillation of the Schiff base linkage on helix H3 and from the force on Arg177 that mediates stability of the retinal in the dark-state of the receptor. The ligand- induced force on Glu113 and Gly114 is disseminated through the receptor as a torsional oscillation that promotes fluctuations of Gly89 – 90 on helix H2 and Gly120 on helix H3. Analogously, the force on Arg177 induces oscillations in the β3 loop of extracellular loop 2 (EL2) that creates a counter-torque from that which is produced from the retinal force on residues 113 and 114. Hence, the propagation of internal stress reveals a network of highly correlated fluctuations that induces a receptor-wide torque-induced rotational motion that resembles the dominant FCA mode uncovered from the MD simulation of the dark-state receptor in Figure 2d.

A secondary force-induced propagation pathway in the dark-state WT receptor is also uncovered in Supplementary Figure S2d. In this case, a transient fluctuation of the retinal intermittently modifies the ligand-receptor interaction such that the interaction with the C9- methyl group of the retinal has a much stronger interaction with Gly120 and Gly121 in helix H3. The fluctuation-induced modification in the ligand-receptor interactions also creates a counter torque centered at Gly174 in EL2. Together the retinal-induced correlated structural fluctuations are translated as an elongation motion that extends from the ligand-binding pocket into the direction of the G-protein binding, which consequently slightly alters the packing in the receptor hydrophobic core and mirrors the secondary FCA mode uncovered in the analysis of the conformational dynamics of the dark-state WT receptor in Figure 2d.

Section 2: Conformational substate probability and transition pathways

A. WT monomer (dark-state)

The identified principal modes from the FCA were used to compute the Gibbs free energy landscape in the conformational space of the individual receptor using gmx sham in gromacs. The conformational states within the minimum of the energy wells labeled as states A, B, and C in Supplementary Figure S3 serve as centroids for clustering with greedy optimization (15) of modularity based on random starting group labels (16). In the figure, state A represents

the dark-state like dynamics of the receptor, state C the active-state like dynamics, and state B illustrates the transition dynamics associated with barrier crossing. The percentages next to each

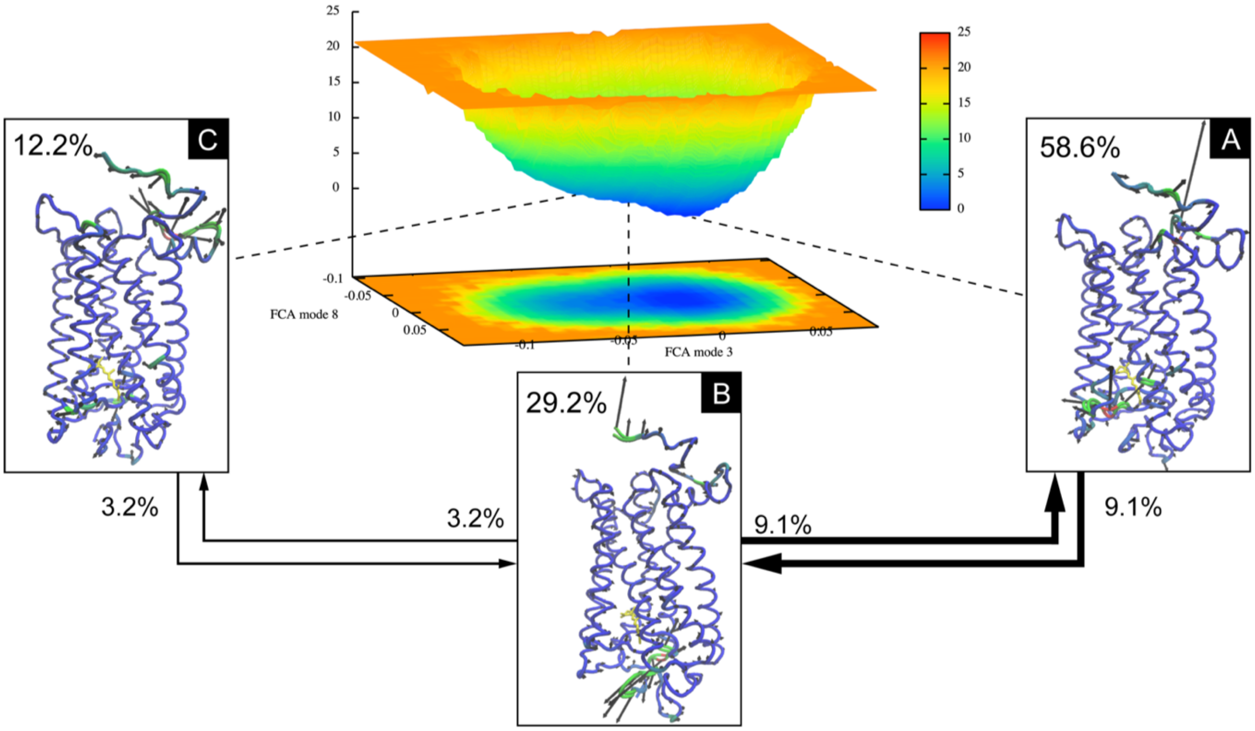

Supplementary Figure S3: Conformational landscape from the FCA of WT (monomer) dark-state rhodopsin with conformational states labeled as A, B, and C, the MD time spent in each state shown as a percentage next to the state, and the transition probability from one state to another labeled with arrows .

conformational structure represent the time the simulation is in each state, i.e. 58.6% of the time the receptor is in state A, etc. The arrows show the transition probability of two consecutive simulation steps based on all of MD simulation steps in the trajectory. For instance, 9.1% of the MD trajectory time, the system is in state A and in state B in the next step. The chance of jumping between state A and C is below 0.1%. From our analysis it becomes clear that the receptor spends most of the time in the dark-like state but samples active-like state dynamics even when not in an active-state.

B. WT H1/H2/H8 – H1/H2/H8 homodimer (dark-state)

Similar to the analyses conducted for the WT monomer, the identified principal modes from the FCA were used to compute the free energy landscape of monomer 1 (the active monomer) of the WT H1/H2/H8 – H1/H2/H8 inactive-state homodimer. In Supplementary Figure S4, the conformational states labeled as A, B, and C represent the dark-state dynamics of the monomer, the active-state-like dynamics of the monomer, and a new state associated with the coupling of the monomers making up the dimer, respectively. The transition probability analysis reveals that like the WT monomer, monomer 1 of the WT dimer spends most of the simulation time in the dark-like state and frequently samples the active-like conformational state. Interestingly, although infrequent, the dimer coupling state (state C) can be sampled from either the dark-like

conformational state (state A) or the active-like conformational state (state B) but has a slightly higher weighting from the active-like conformational state.

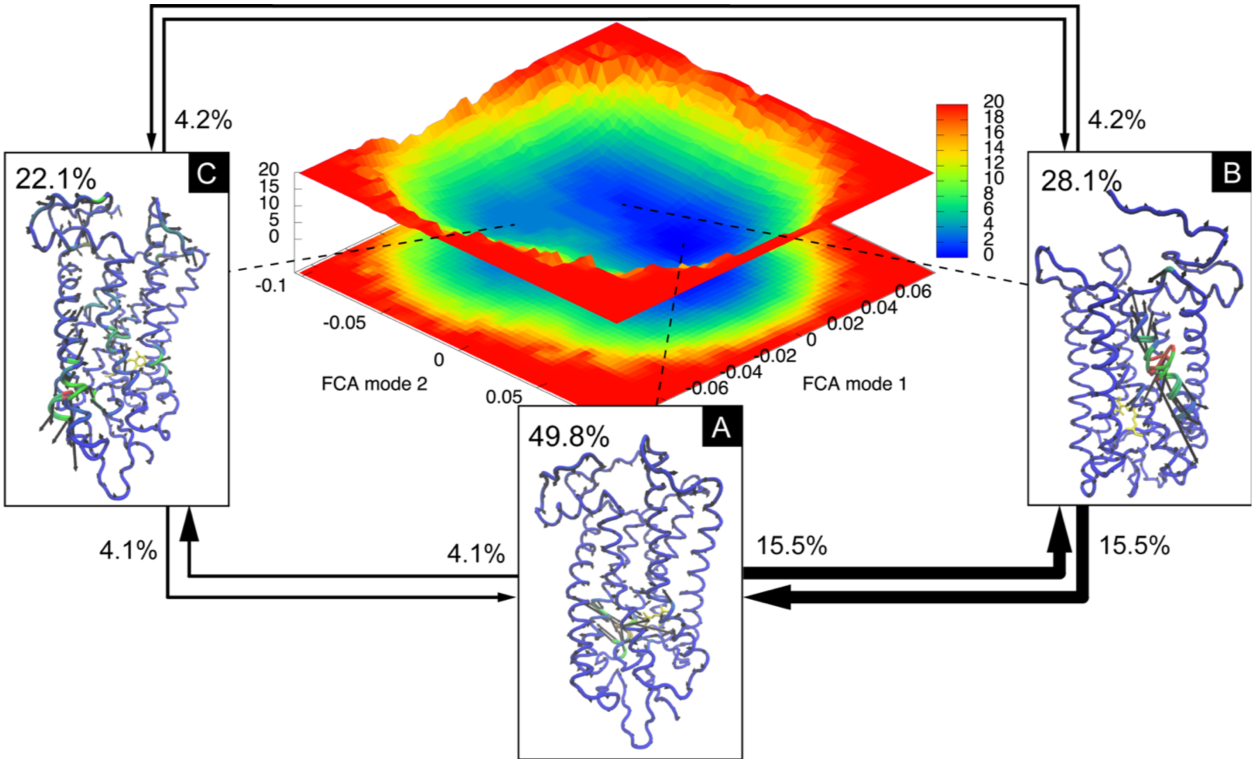

Supplementary Figure S4: Conformational landscape from the FCA of ***monomer 1*** of the inactive-state WT H1/H2/H8 – H1/H2/H8 homodimer where conformational states are labeled as A, B, and C, the MD time spent in each state shown as a percentage next to the state, and the transition probability from one state to another is labeled with arrows.

C. P23H H4/H5 – H4/H5 homodimer (dark-state)

A analogous analysis with the P23H H4/H5 – H4/H5 inactive-state homodimer in Supplementary Figure S5 uncovers surprising details about the sampling of conformational states in the energy landscape of the dimerized receptor. Particularly, monomer 1 of the dimer has four distinct states labeled as A, B, C, and D in Supplementary Figure S5. States A and B represent the dark-like and active-like states, respectively as observed in the WT monomer receptor in Figure 3. States C and D are new functional states in the P23H dimer conformational landscape that can be described as a transient cavity in the EC region of helices H5 and H6 (state C) and an axial elongation torsion (state D). Interestingly, the analysis reveals that although state C is not the lowest energy conformational state in the P23H dimer energy landscape, the receptor spends the majority of time in this state and most frequently makes transitions from state C to state D. Hence, these results reveal that this particular P23H homodimer configuration/interactions plays a pivotal role in shifting the functional dynamics of the receptor from rhodopsin-like to something distinctively different.

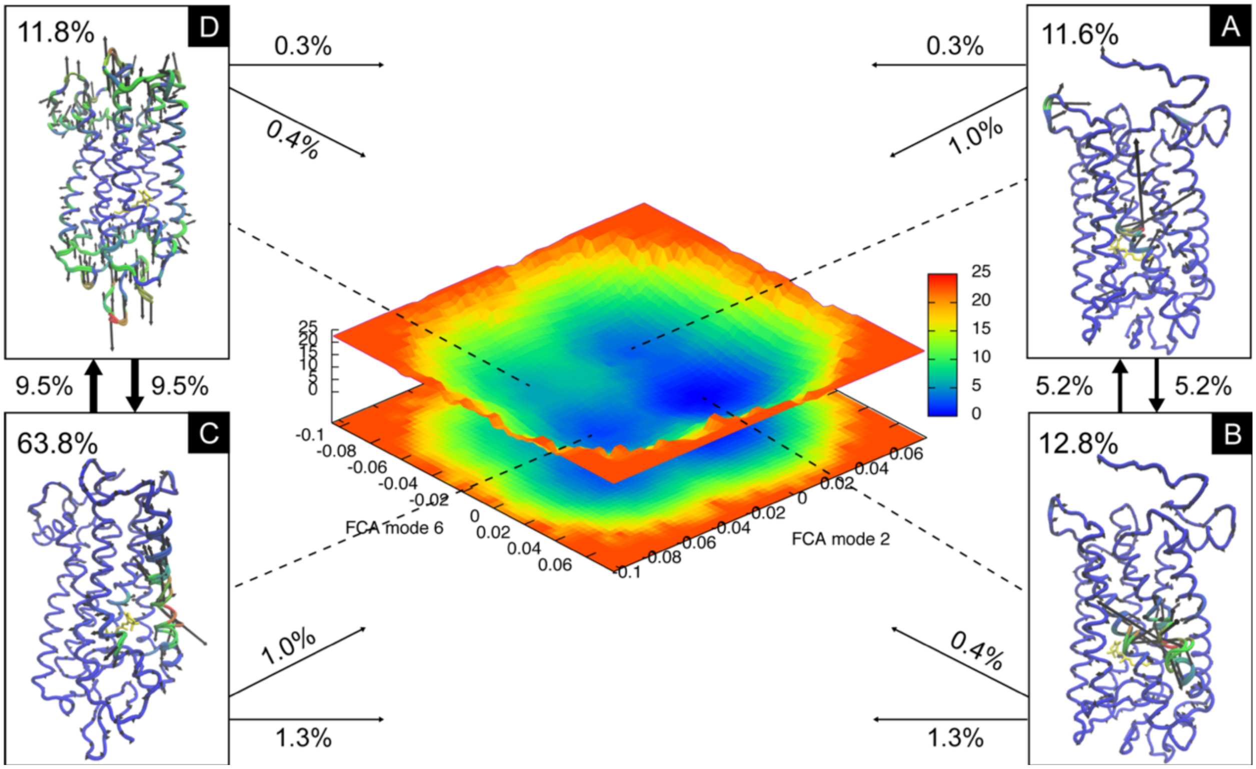

Supplementary Figure S5: Conformational landscape from the FCA of ***monomer 1*** of the P23H H4/H5-H4/H5 dark-state homodimer. The conformational states are labeled as A, B, C, and D. The MD time spent in each state is shown as a percentage next to the conformational state, and the transition probability is illustrated by an arrow to indicate the direction of transition and a percentage to illustrate the probability of transition.

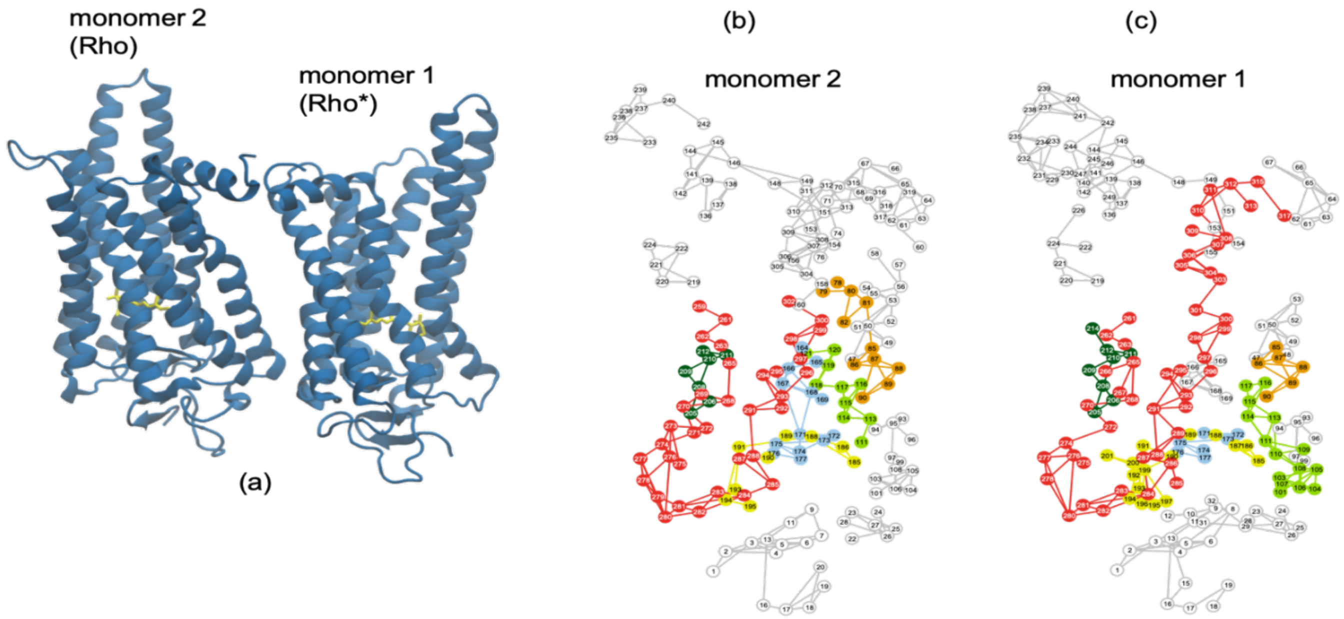

Supplementary Figure S6: (**a**) Carton representation of rhodopsin molecules illustrating the arrangement of the receptors in the WT Meta-II H1/H2/H8 – H1/H2/H8 homodimer configuration. Rho* refers to the active monomer within the dimer. (**b**) and (**c**) show the 2-D mapping of the LSF from monomer *2* and monomer 1, respectively.

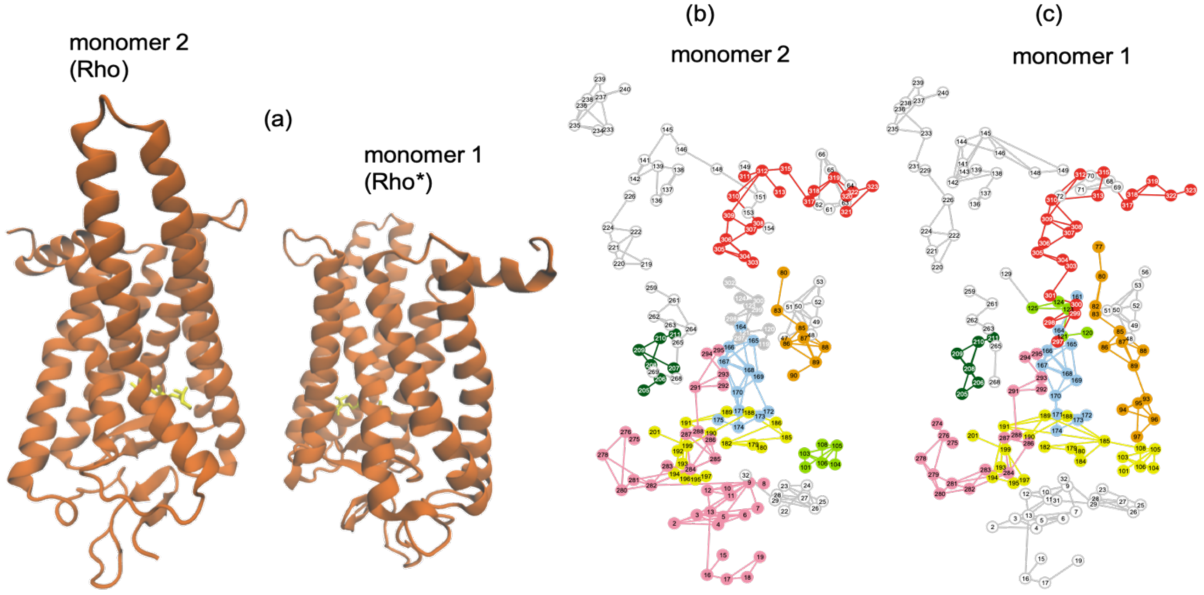

Supplementary Figure S7: (**a**) Carton representation of rhodopsin molecules illustrating the arrangement of the receptors in the WT Meta-II H4/H5 – H4/H5 homodimer. (**b**) and (**c**) show the 2-D mapping of the LSF from monomer 1 and monomer 2 in the H4/H5 – H4/H5 homodimer, respectively.

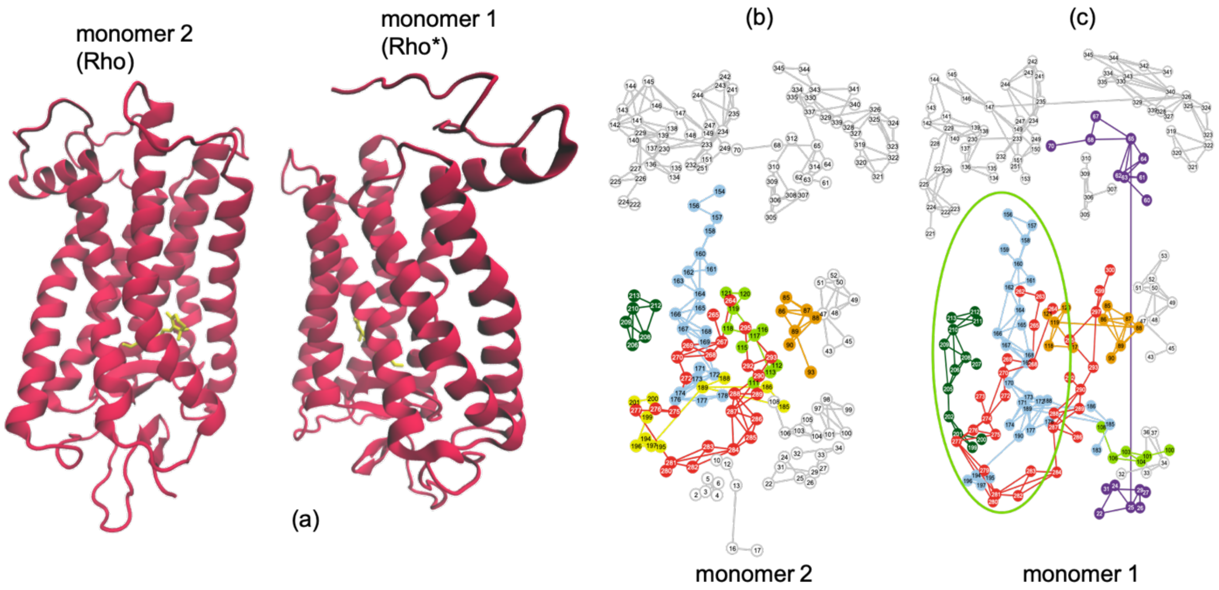

Supplementary Figure S8: (**a**) Carton representation of rhodopsin molecules illustrating the arrangement of the receptors in the P23H dark-state H4/H5 – H4/H5 dimer configuration. (**b**) and (**c**) show the 2-D mapping of the LSF from monomer 2 and monomer 1 in the dark-state H4/H5 – H4/H5 homodimer, respectively.

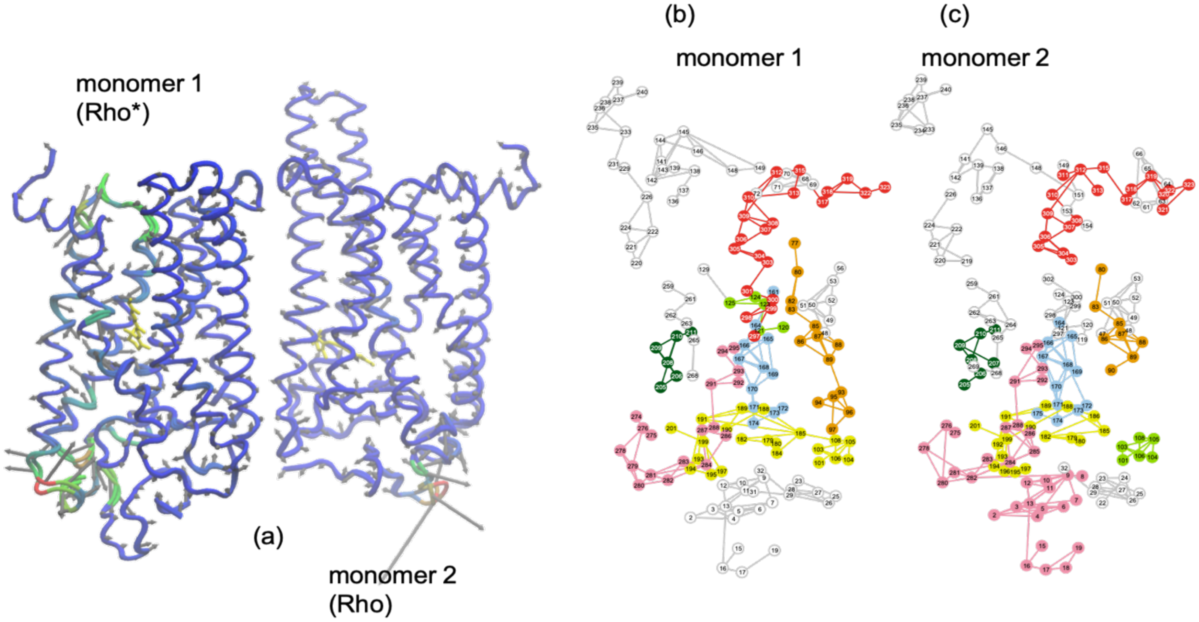

Supplementary Figure S9: C-α representation of the dominant PCA mode (PCA1) of (**a**) the monomers making up the Meta-II-P23H H4/H5 – H4/H5 homodimer and (**b**) and (**c**) the corresponding 2-D mapping of the LSF from monomer 1 and monomer 2 , respectively in the Meta-II-P23H H4/H5 – H4/H5 homodimer.

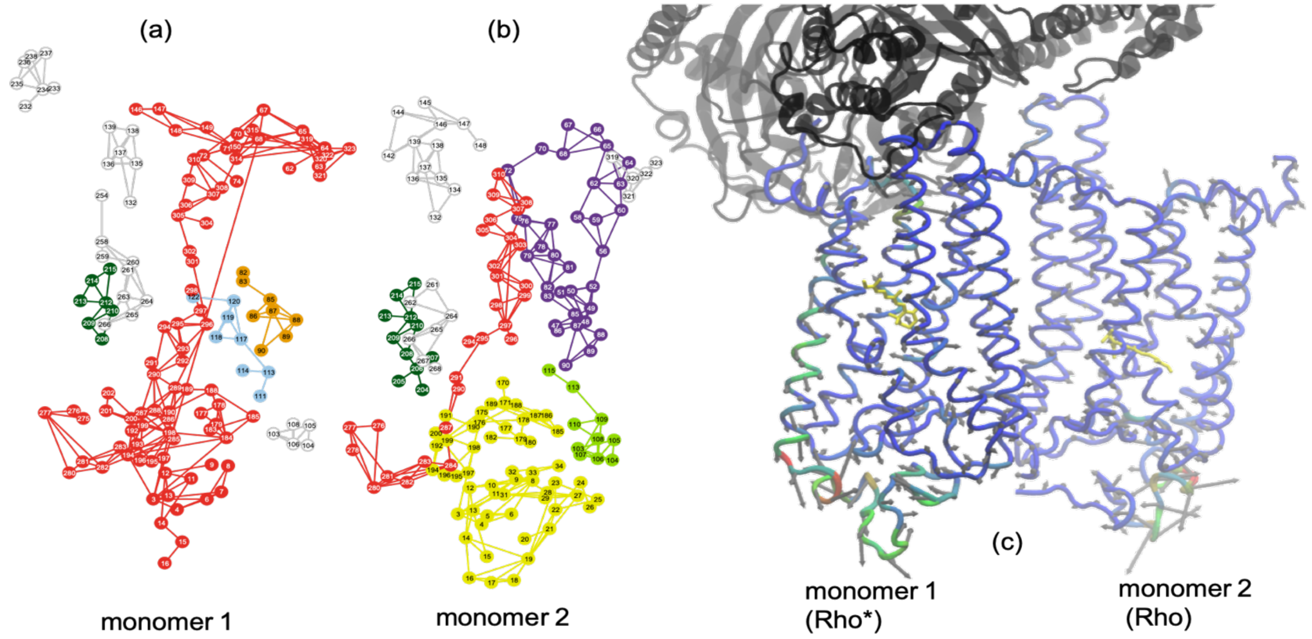

Supplementary Figure S10: : The 2-D mapping of the LSF of (**a**) monomer 1 and **(b**) monomer 2 of the Meta-II-P23H H4/H5 – H4/H5 dimer bound with the G-protein transducin. (**c**) C-α representation of the dominant PCA mode of the monomers making up the Meta-II P23H H4/H5 – H4/H5 G-protein bound dimer.

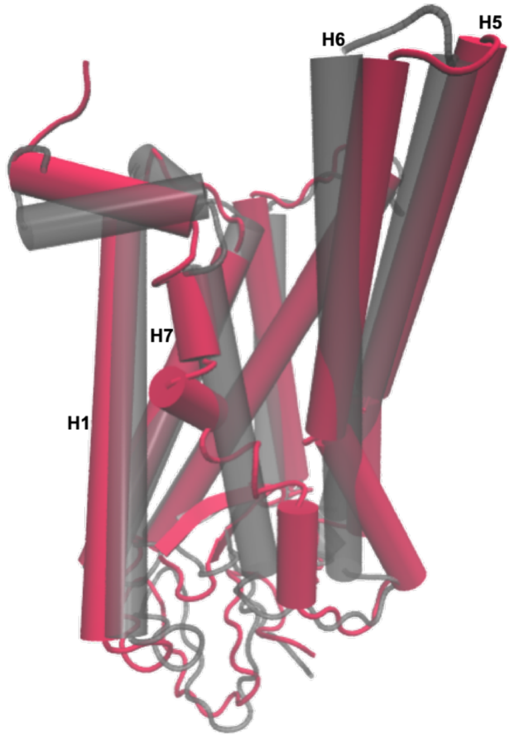

Supplementary Figure S11: A structural comparison of Meta-II rhodopsin bound with the G-protein (gray, transparent) and monomer 1 of the Meta-II-P23H H4/H5 – H4/H5 homodimer (red) when bound with the G-protein

Section 3: General findings about rhodopsin dimer interactions as determined from MD simulation

A. *Rhodopsin homodimer inter-receptor binding interactions and rhodopsin signaling-state dynamics*

In all of the simulations on rhodopsin dimers conducted, we determined that dimers formed with strong inter-receptor interactions feature one active monomer within the dimer while the other monomer within the dimer remained in an inactive-state. In our interpretation of the active- or signaling state (Meta-II), the rhodopsin molecule supports an inter-residue network of interactions that connect dynamics in the retinal-binding site with dynamics in the intracellular G-protein binding region (Supplementary Figure S6 and Figure 4c). On the other hand, the inactive receptor features intra-helical H-bonds and hydrophobic packing interactions (such as the H-bonds linking helix H3 and helix H5 involving Glu122 and His211 and the hydrophobic packing interactions between helix H3 and helix H6 connecting Gly121, Leu125, Phe261, and Trp265) that hinder helical rearrangements that are necessary for the receptor to adopt a stable active conformation (Supplementary Figure S6). We have also uncovered that rhodopsin dimers with weaker monomer-monomer inter-residue interactions, such as the lipid-supported H4/H5 – H4/H5 dimer examined in the main section of this manuscript, behave more like single (un-dimerized) receptors (Supplementary Figure S7 and Figures 7a-c).

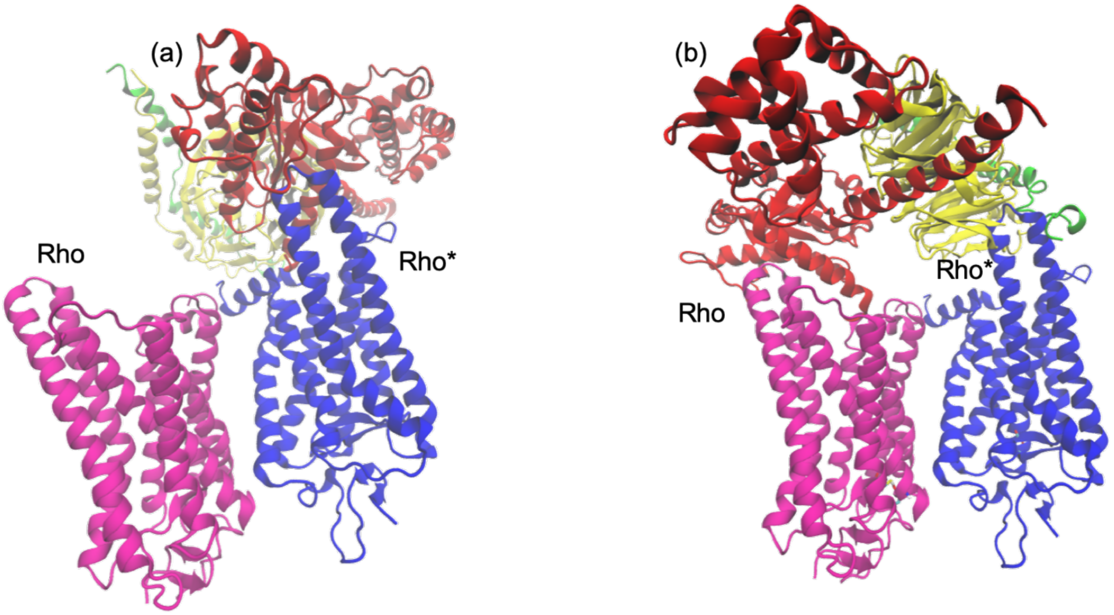

Supplementary Figure S12: : An illustration of two different models for G-protein binding in the H1/H2/H8 – H1/H2/H8 Meta-II dimer. In the figures, Rho* refers to the active monomer within the dimer and Rho the inactive monomer. In (**a**) The G-protein is bound to only one monomer in the dimer which we will refer to as **configuration 1** and in (**b**) the G-protein makes contact with both monomers making up the dimer – **configuration 2**.

B. G-protein binding orientation in rhodopsin homodimers

When preparing the MD simulations of rhodopsin dimers forming a complex with the heterotrimeric G-protein transducin, it was unclear how the G-protein should/could be oriented on the dimer intracellular surface. For instance, the G-protein complex can make contact with both monomers of the dimer as shown in Supplementary Figure S12b or alternatively binds to only one of the monomers in the dimer complex as displayed in Supplementary Figure S12a. We found a clear orientation preference for the G-protein in the dimers with strong inter-residue monomer association (See Supplementary section, *Dimer inter-receptor binding interactions and rhodopsin signaling-state dynamics*) and evidence that the specific binding of the G-protein to the rhodopsin dimer has a strong influence on the functional and structural dynamics of the complex as a whole. For instance, in the case where the G-protein makes contact with both monomers comprising the dimer, we detect an overall fragmentation of the inter-protein dynamics of the receptor in direct contact with the C-terminal region of the G_α - subunit (Supplementary Figure S13c). Interestingly, this type of widespread fragmentation of inter-protein interactions was also observed in an earlier study in which we conducted MD simulations to study the dynamics of Meta-II when complexed with the protein arrestin[11]. For the rhodopsin monomer in direct contact with the G_β and $G\gamma$ subunits of the G-protein, we observe a destabilization of the intra-residue pathway of interactions forming the signaling pathway from the retinal-site to the G-protein binding region (Supplementary Figure S13d). In summary, in the G-protein configuration in which both monomers make contact with the G-protein, we observe an overall desensitization of signaling in the active-state (strong-interacting) dimer. Alternatively, in the G-protein dimer configuration in which the G-protein binds to only one monomer within the dimer we observe a stabilization of the receptor signaling state (Supplementary Figure S13b) in the bound monomer. The unbound monomer in this configuration features a network of interactions (Supplementary Figure S13a) that support intra-

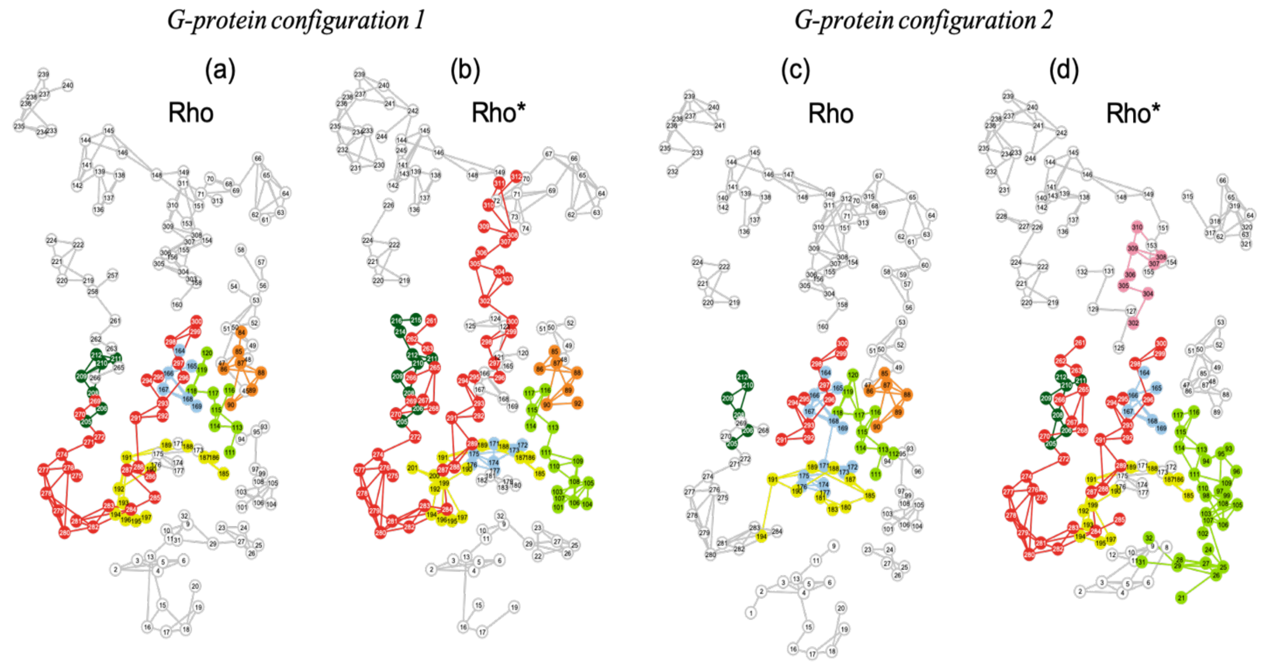

Supplementary Figure S13: The 2-D LSF of (**a**) Rho and (**b**) Rho* from **configuration 1** of G-protein binding in the H1/H2/H8 – H1/H2/H8 homodimer from Supplementary Figure S14 and **(c)** Rho and (**d**) Rho* from **configuration 2**.

helical H-bonds and hydrophobic packing interactions that resemble the inactive-state receptor. Our findings are in line with a previous EM study on dimeric states of rhodopsin bound with the G-protein transducin (17) that uncovered that photoactivated dimeric rhodopsin is structurally and functionally in an asymmetric state in the Rho*-G_t complex.

C. WT-P23H heterodimerization and dimer (dys)function

We have also conducted simulations in which we have created heterodimeric complexes comprised of WT and P23H rhodopsin monomers in different dimeric configurations. Although, we have focused our analyses primarily on the H1/H2/H8 – H1/H2/H8 and H4/H5 – H4/H5 dimer configurations that were discussed extensively in the main section of this manuscript and were also found to be the most prominent homodimer configurations formed in rhodopsin. In both heterodimer configurations, we observed a deleterious effect on the functional dynamics of the WT receptor within the complex and little change in the functional dynamics of the P23H monomer within the complex. Explicitly, in the H1/H2/H8 – H1/H2/H8 dimer (Supplementary Figure S14), the formation of the Meta-II P23H – WT heterodimer resulted in strong interaction between the N-terminus of the P23H monomer and the WT monomer H3 core residues (Ala117 – Leu125) in the dimer as seen in Supplementary Figures S14a-b. The helix H3 core residues have a stabilizing effect on the Cys110-Cys187 disulfide bond and hence, the overall stability of the ECD of the WT receptor. Coincidentally, the strong interaction in the EC region of the heterodimer results in a wider, highly destabilized ECD and an extended EC retinal-binding pocket that severely decreases the interaction between the retinal and the WT receptor, particularly in the EC region of helix H6 that normally supports essential interactions with the β–ionone ring of the retinal in the WT monomer. Similarly, in the H4/H5 – H4/H5 heterodimer (Supplementary Figure S15) we observe a severe distortion of the ECD of the WT receptor when complexed with P23H. In this case, the extreme unfolding/unstructured region of the H4/H5-

H4/H5 EC region of the P23H monomer (within the dimer) creates strong interactions with the EC region of

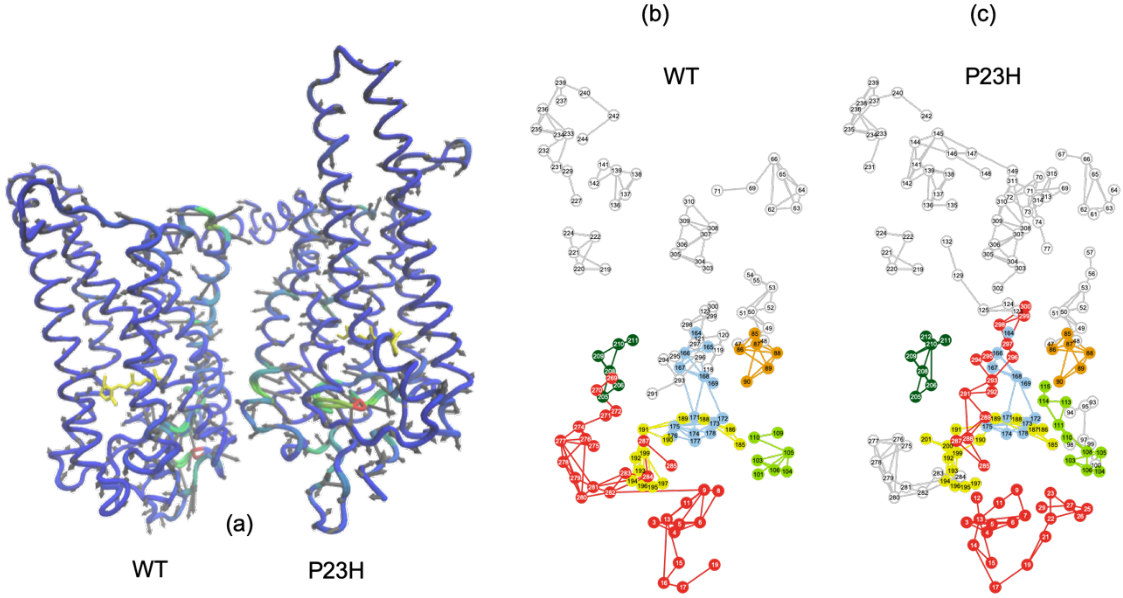

Supplementary Figure S14: C-α representation of PCA1 of (**a**) the monomers making up the Meta-II WT – P23H H1/H2/H8 – H1/H2/H8 **heterodimer** and (**b**) and (**c**) the corresponding 2-D mapping of the LSF from the WT monomer and the P23H monomer, respectively that form the heterodimer.

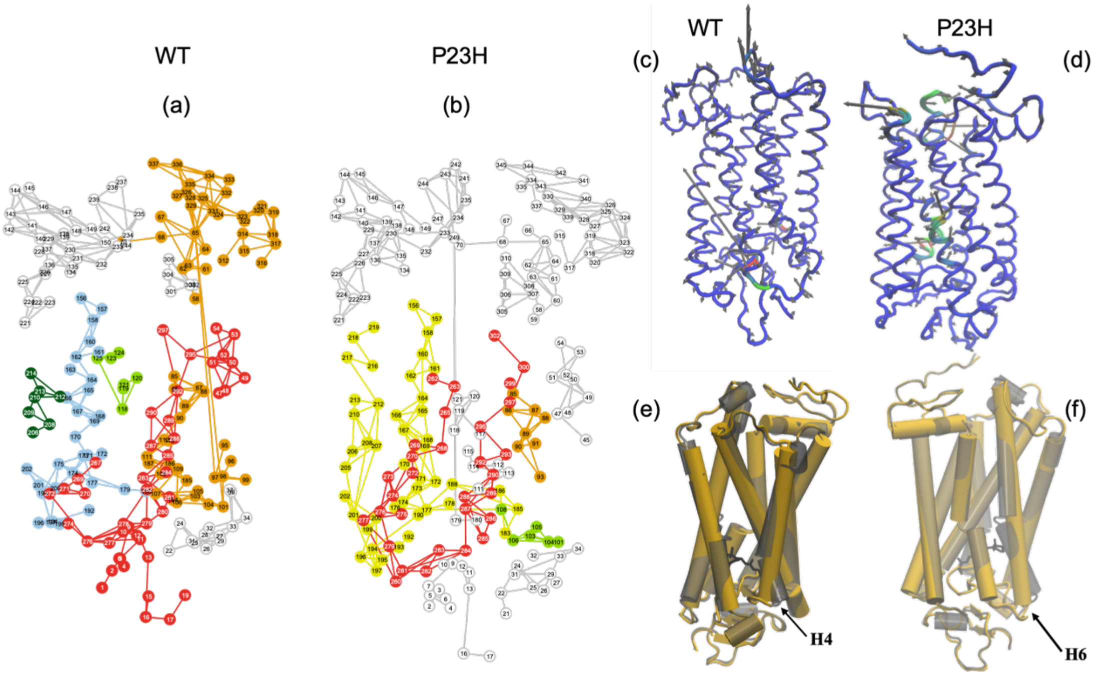

Supplementary Figure S15: The 2-D mapping of the LSF from the (**a**) WT monomer and (**b**) the P23H monomer from the dark-state P23H – WT H4/H5 – H4/H5 **heterodimer** and (**c**) and (**d**) the corresponding PCA1 of the WT and P23H monomer, respectively that form the heterodimer. In (**e**) and (**f**) an overlay of a C-α cartoon representation of WT rhodopsin (orange) with

the WT monomer comprising the heterodimer (transparent gray) demonstrate the extreme structural changes, particularly in the ECD, that take place in the WT receptor when bound with the P23H monomer in the heterodimer.

helices H4 and H5 of the WT monomer. This, coupled with the weaker interactions at the IC side involving the H4 and H5 regions of the both receptors results in the extreme distortion of the entire tertiary structure of the WT receptor (Supplementary Figures S15e – f). Particularly, the EL2 region of the WT receptor is strongly shifted toward the P23H monomer while a counter-distortion is created that shifts helix H1 of the WT receptor closer to helix H8 in the same receptor. As a result, a large cavity is dynamically created in the ECD of the WT receptor that alternatively shifts EL2 and EL3 in extreme opposite directions and then forcefully compresses EL2 through the receptor hydrophobic core. In summary, analogous to the H1/H2/H8 – H1/H2/H8 P23H - WT heterodimer - the dark-state H4/H5 – H4/H5 heterodimer fully destabilizes the ECD of the WT receptor and presumably also eliminates the functional dynamics of not only the WT receptor but the entire heterodimer as a whole.

Section 4: Allosteric changes in Meta-II-P23H and G-protein binding and interactions
In addition to studying the active-state receptor, we have also used MD simulation as a tool to guide us in understanding the potential differences in the distinct Meta-II sequences when complexed with the G-protein transducin (G_T). Our findings suggest that allosteric changes in the structure and long-range interactions in Meta-II-P23H are the source of the principal variances that we identify when contrasting the G-protein bound state with that of the WT complex (Supplementary Figure S16). Both direct contact sites and the extended interactions that form the rhodopsin/G-protein interface are considerably modified in Meta-II-P23H. The C-terminus of the G_α-subunit of the G-protein forms the main contact site with rhodopsin at helix H7/IL3. Using MD simulations to monitor the weak interactions at the rhodopsin/G-protein interface, we ascertain that disrupted intra-residue interactions on the cytoplasmic side of TM7 in Meta-II-P23H (Supplementary Figure S16b) lead to weaker contact interactions between Meta-II-P23H IL3 and the G_α- C-terminus (when contrasted with the WT receptor). The reason for the alteration in rhodopsin/G-protein C-terminus interactions is from the increased flexibility of the IC side of helix H7 due to the (*i*) disruption of the NPxxY microdomain and (*ii*) an overall decrease in the number of water molecules in the solvation shell in Meta-II-P23H (Supplementary Figure S17). As a consequence, both IL3 and cytoplasmic side of helix H7 are tilted away from the transmembrane helical core and helix H7 is shifted slightly closer to the EC side of the receptor. In addition to contributing to a reduced hydrophobic contact area, these slight structural changes lead to the loss of conserved H-bonds between the receptor and the G-protein (18). For instance, the hydrogen-bond between Gln237 (IL3) and Asp334 (G_α-C-terminus) is lost in the Meta-II-P23H/G-protein complex (Supplementary Figure S16b). Additionally, our analysis indicates that the diminished Meta-II-P23H solvation shell also leads to the loss of the conserved solvent-mediated H-bond connection between Thr243 (IL3) and Asp331 (G_α-C-terminus).

Besides the C-terminus, other regions that have been reported to be important for receptor/G-protein engagement are also noticeably altered in the Meta-II-P23H/G-protein complex. Comparison with the WT/G-complex structure in Supplementary Figure S16b highlights that the conserved contact site formed by the network of solvent-mediated H-bonds (19) involving Lys311 (helix H8) and residues residing on α_3/α_4 helices of the Ras-like domain (Asp307 and Phe226) on the G_α– subunit are also missing in the Meta-II-P23H complex. We also observe a

decrease in the contact area involving rhodopsin IL2 and the helical domain of G_α. Hydrophobic packing within the rhodopsin P23H transmembrane core (mostly involving TM3 - TM5) coupled with EL2 deformation, shifts the IL2 further to the EC side of the receptor and also imparts a slight outward tilt to the loop. The structural modification in the IL2 conformation combined with the receptor's diminished solvation shell moves the helical domain of the G_α-subunit out of reach for rhodopsin intracellular loop contact. Particularly, we detect the loss of the solvent-supported H-bond interaction (20) involving Asp145 (IL2) with Val101 and Pro102 on the G_α-helical domain. Rhodopsin interaction with residues in the Ras-like domain of G_α have been reported to be associated with G-protein specificity (21). Whereas rhodopsin interaction with the helical domain of the G_α-subunit has been implicated in enhancing the efficiency of G-protein signaling (22). The differences that we identify in the Meta-II-P23H/G-protein complex suggests that it is likely that the mutant receptor G-protein complex would have a substantially reduced efficiency when compared with its WT counterpart.

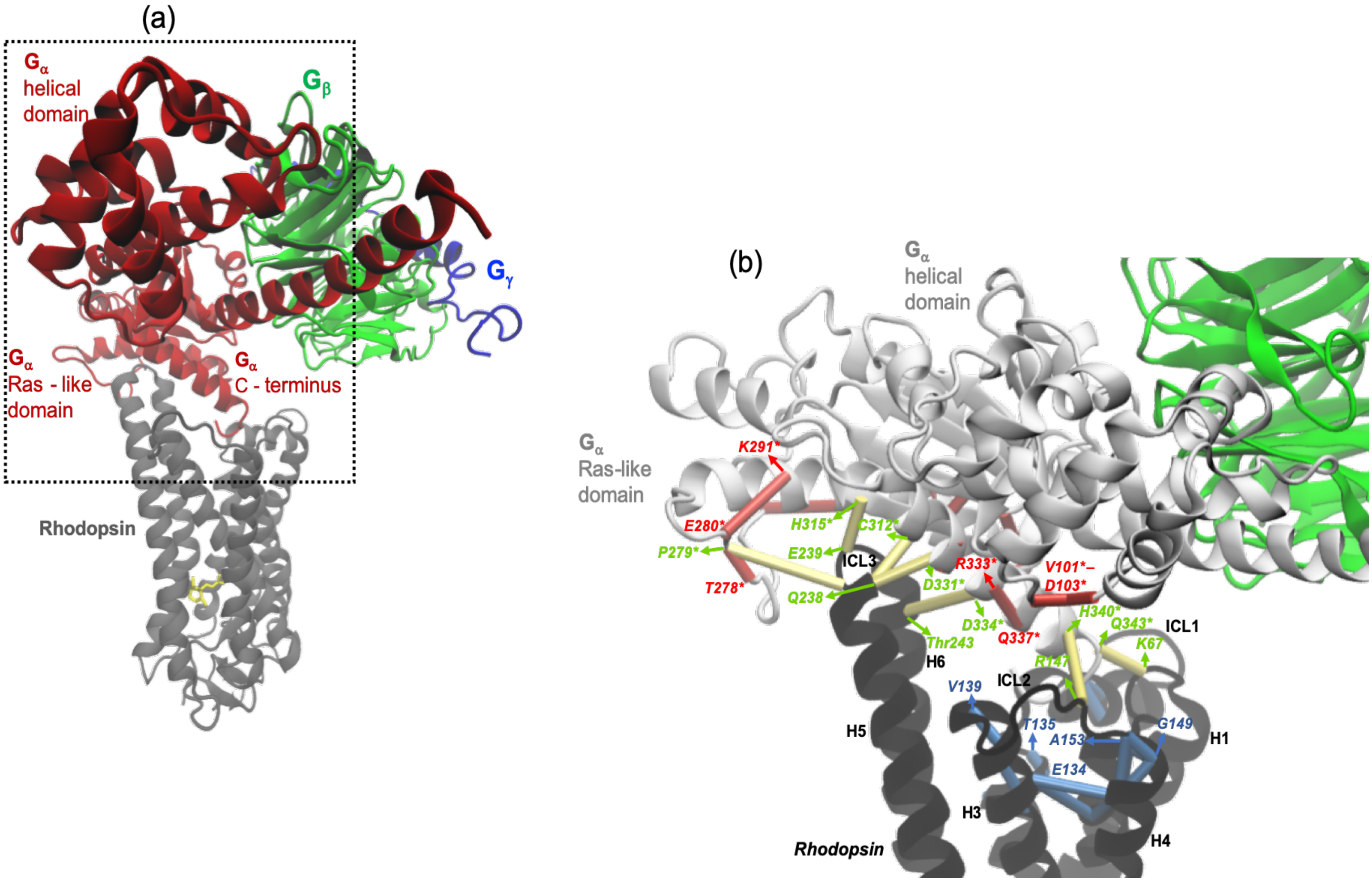

Supplementary Figure S16: (**a**) Cartoon representation of Meta-II rhodopsin bound to the G-protein transducin. (**b**) Cartoon representation of the Meta-II P23H bound G-protein complex displaying rhodopsin-intra-protein H-bonds (blue), G-protein intra-protein H-bonds (red), and Meta-II/G-protein inter-protein H-bonds (yellow) that are absent in the mutant complex when compared with the WT/G-protein complex. An * next to the residue label refers to residues in the G-protein complex.

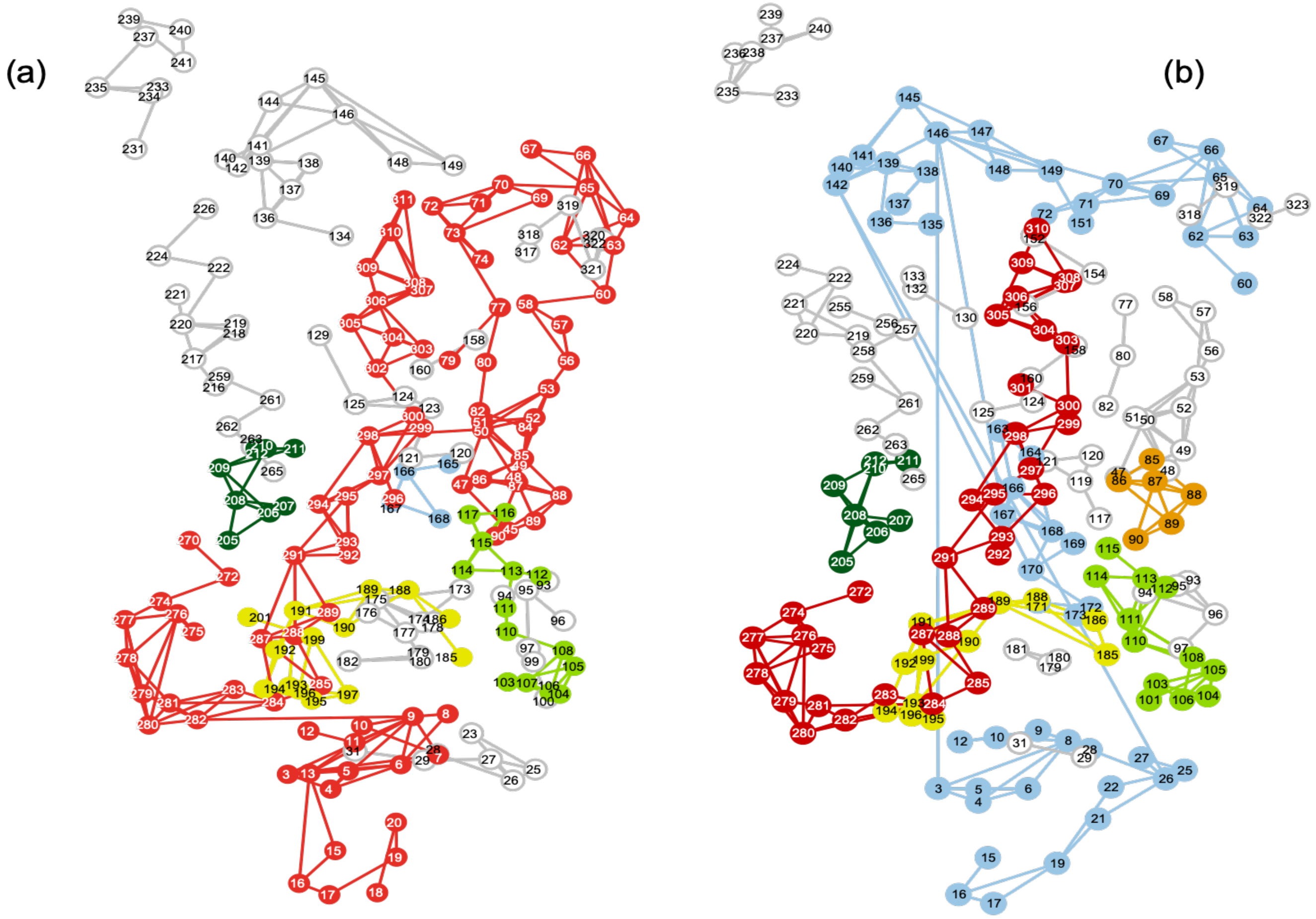

Supplementary Figure S17: A 2-D mapping of the LSF from (**a**) Meta-II bound with the G-protein transducin and (**b**) Meta-II P23H bound with transducin.

References

1. D. D. Oprian, R. S. Molday, R. J. Kaufman, H. G. Khorana, Expression of a synthetic bovine rhodopsin gene in monkey kidney cells. *Proc. Natl. Acad. Sci. U. S. A.* **84**, 8874–8878 (1987).

2. P. J. Reeves, J.-M. Kim, H. G. Khorana, Structure and function in rhodopsin: a tetracycline-inducible system in stable mammalian cell lines for high-level expression of opsin mutants. *Proc. Natl. Acad. Sci. U. S. A.* **99**, 13413–13418 (2002).

3. S. M. Noorwez, *et al.*, Retinoids Assist the Cellular Folding of the Autosomal Dominant Retinitis Pigmentosa Opsin Mutant P23H. *J. Biol. Chem.* **279**, 16278–16284 (2004).

4. J. Mitchell, *et al.*, Comparison of the molecular properties of retinitis pigmentosa P23H and N15S amino acid replacements in rhodopsin. *PLOS ONE* **14**, e0214639 (2019).

5. J. Hwa, P. J. Reeves, J. Klein-Seetharaman, F. Davidson, H. G. Khorana, Structure and function in rhodopsin: Further elucidation of the role of the intradiscal cysteines, Cys-110, -185, and -187, in rhodopsin folding and function. *Proc. Natl. Acad. Sci. U. S. A.* **96**, 1932–1935 (1999).

6. K. Fahmy, *et al.*, Protonation states of membrane-embedded carboxylic acid groups in rhodopsin and metarhodopsin II: a Fourier-transform infrared spectroscopy study of site-directed mutants. *Proc. Natl. Acad. Sci. U. S. A.* **90**, 10206–10210 (1993).

7. A.-N. Bondar, M. Knapp-Mohammady, S. Suhai, S. Fischer, J. Smith, Ground-State Properties of the Retinal Molecule: from Quantum Mechanical to Classical Mechanical Computations of Retinal Proteins. *Theor. Chem. Acc. Theory Comput. Model. Theor. Chim. Acta* **130**, 1169–1183 (2011).

8. H. J. C. Berendsen, J. P. M. Postma, W. F. van Gunsteren, A. DiNola, J. R. Haak, Molecular dynamics with coupling to an external bath. *J. Chem. Phys.* **81**, 3684 (1984).

9. B. Hess, H. Bekker, H. J. C. Berendsen, J. G. E. M. Fraaije, LINCS: A linear constraint solver for molecular simulations. *J. Comput. Chem.* **18**, 1463–1472 (1997).

10. U. Essmann, *et al.*, A smooth particle mesh Ewald method. *J. Chem. Phys.* **103**, 8577–8593 (1995).

11. O. F. Lange, H. Grubmüller, Full correlation analysis of conformational protein dynamics. *Proteins* **70**, 1294–1312 (2008).

12. M. D. Daily, J. J. Gray, Local motions in a benchmark of allosteric proteins. *Proteins* **67**, 385–399 (2007).

13. A. Pandini, A. Fornili, F. Fraternali, J. Kleinjung, GSATools: analysis of allosteric communication and functional local motions using a structural alphabet. *Bioinformatics* **29**, 2053–2055 (2013).

14. W. Stacklies, C. Seifert, F. Graeter, Implementation of force distribution analysis for molecular dynamics simulations. *BMC Bioinformatics* **12**, 101 (2011).

15. M. E. J. Newman, M. Girvan, Finding and evaluating community structure in networks. *Phys. Rev. E* **69**, 026113 (2004).

16. R. L. Breiger, S. A. Boorman, P. Arabie, An algorithm for clustering relational data with applications to social network analysis and comparison with multidimensional scaling. *J. Math. Psychol.* **12**, 328–383 (1975).

17. B. Jastrzebska, T. Orban, M. Golczak, A. Engel, K. Palczewski, Asymmetry of the rhodopsin dimer in complex with transducin. *FASEB J.* **27**, 1572–1584 (2013).

18. V. Katritch, V. Cherezov, R. C. Stevens, Structure-Function of the G-protein-Coupled Receptor Superfamily. *Annu. Rev. Pharmacol. Toxicol.* **53**, 531–556 (2013).

19. A. I. Kaya, *et al.*, A Conserved Hydrophobic Core in Gαi1 Regulates G Protein Activation and Release from Activated Receptor. *J. Biol. Chem.* **291**, 19674–19686 (2016).

20. P. A. Hargrave, H. E. Hamm, K. P. Hofmann, Interaction of rhodopsin with the G-protein, transducin. *BioEssays News Rev. Mol. Cell. Dev. Biol.* **15**, 43–50 (1993).

21. N. V. Eps, *et al.*, Gi- and Gs-coupled GPCRs show different modes of G-protein binding. *Proc. Natl. Acad. Sci.*, 201721896 (2018).

22. J. P. Mahoney, R. K. Sunahara, Mechanistic insights into GPCR-G protein interactions. *Curr. Opin. Struct. Biol.* **41**, 247–254 (2016).
